# PRRC2 proteins impact translation initiation by promoting leaky scanning

**DOI:** 10.1093/nar/gkad135

**Published:** 2023-03-03

**Authors:** Jonathan Bohlen, Mykola Roiuk, Marilena Neff, Aurelio A Teleman

**Affiliations:** German Cancer Research Center (DKFZ), 69120 Heidelberg, Germany; CellNetworks - Cluster of Excellence, Heidelberg University, Germany; Heidelberg University, 69120 Heidelberg, Germany; Heidelberg Biosciences International Graduate School (HBIGS), Germany; National Center for Tumor Diseases (NCT), partner site, 69120 Heidelberg, Germany; Laboratory of Human Genetics of Infectious Diseases, Necker Branch, Institut National de la Santeé et de la Recherche Meédicale U1163, Paris, France; University of Paris, Imagine Institute, Paris, France; German Cancer Research Center (DKFZ), 69120 Heidelberg, Germany; CellNetworks - Cluster of Excellence, Heidelberg University, Germany; Heidelberg University, 69120 Heidelberg, Germany; National Center for Tumor Diseases (NCT), partner site, 69120 Heidelberg, Germany; German Cancer Research Center (DKFZ), 69120 Heidelberg, Germany; Heidelberg University, 69120 Heidelberg, Germany; German Cancer Research Center (DKFZ), 69120 Heidelberg, Germany; CellNetworks - Cluster of Excellence, Heidelberg University, Germany; Heidelberg University, 69120 Heidelberg, Germany; Heidelberg Biosciences International Graduate School (HBIGS), Germany; National Center for Tumor Diseases (NCT), partner site, 69120 Heidelberg, Germany

## Abstract

Roughly half of animal mRNAs contain upstream open reading frames (uORFs). These uORFs can represent an impediment to translation of the main ORF since ribosomes usually bind the mRNA cap at the 5′ end and then scan for ORFs in a 5′-to-3′ fashion. One way for ribosomes to bypass uORFs is via leaky scanning, whereby the ribosome disregards the uORF start codon. Hence leaky scanning is an important instance of post-transcriptional regulation that affects gene expression. Few molecular factors regulating or facilitating this process are known. Here we show that the PRRC2 proteins PRRC2A, PRRC2B and PRRC2C impact translation initiation. We find that they bind eukaryotic translation initiation factors and preinitiation complexes, and are enriched on ribosomes translating mRNAs with uORFs. We find that PRRC2 proteins promote leaky scanning past translation start codons, thereby promoting translation of mRNAs containing uORFs. Since PRRC2 proteins have been associated with cancer, this provides a mechanistic starting point for understanding their physiological and pathophysiological roles.

## Graphical Abstract

**Graphical Abstract:**
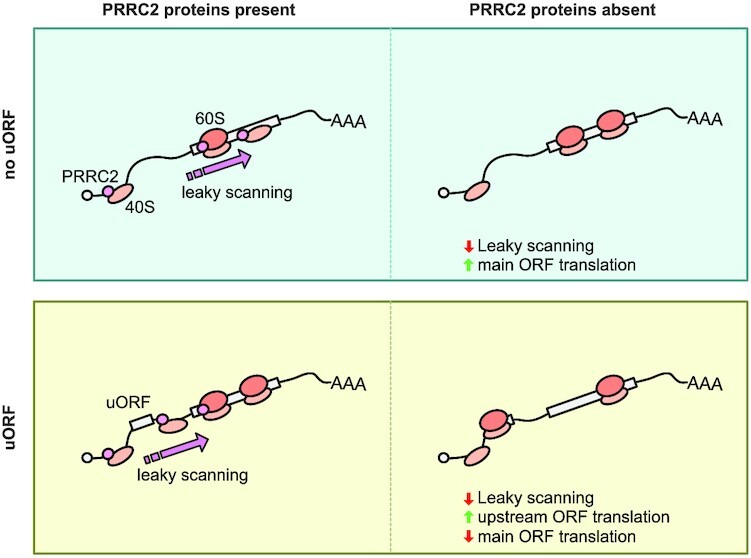
PRRC2A, PRRC2B and PRRC2C proteins impact translation initiation by promoting leaky scanning at the start codons of upstream ORFs (uORFs), overlapping uORFs (oORFs) and main ORFs. In the absence of PRRC2 proteins, translation of mRNAs lacking uORFs or oORFs increases. In contrast, on mRNAs that have high initiation rates on uORFs or oORFs, the absence of PRRC2 proteins causes translation of the uORF or oORF to increase and as a consequence translation of the main ORF is reduced.

## INTRODUCTION

Translation of mRNA into protein, the final step of gene expression, is tightly regulated both because it is energetically expensive, and because it directly results in changes in a cell's proteome, thereby allowing cells to quickly react to environmental changes. Much of translation regulation occurs at the initiation step (1). Translation initiation starts with recruitment to the mRNA cap of the 43S pre-initiation ribosomal complex (PIC), consisting of the small 40S ribosomal subunit bound to the ternary complex (eIF2 + GTP + initiator tRNA) as well as a large number of translation initiation factors including eIF1, eIF1A, eIF4A, eIF4E, eIF4G and the eIF3 complex (for review see (2–4)). The 43S PIC then scans in a 5′ to 3′ direction towards the main Open Reading Frame (ORF). While scanning, the 43S PIC searches for a start codon that is embedded in a favorable sequence context. In roughly half of mammalian mRNAs, however, this first start codon does not belong to the main ORF(mORF), but rather to an upstream ORF (uORF) (5). These uORFs can thereby reduce translation of the main ORF by hijacking the scanning 43S ribosome. Two mechanisms exist to attenuate or circumvent this uORF-mediated repression: reinitiation and leaky scanning. In reinitiation, the ribosome translates the uORF, but after termination it regains an initiator tRNA, resumes scanning towards the main ORF, and then successfully translates the mORF that is further downstream (6). In contrast, in leaky scanning the ribosome bypasses the uORF start codon and continues scanning towards the mORF (7).

We investigate here the family of Proline Rich Coiled-Coil 2 (PRRC2) proteins PRRC2A, PRRC2B and PRRC2C, which are encoded by three separate genes, and whose molecular functions are only starting to be studied. The PRRC2 proteins were previously detected as mRNA interacting proteins in several transcriptome-wide RNA interactome capture screens (8,9) and they were found to interact with the non-canonical initiation factor eIF4G2/NAT1 (10). PRRC2A was shown to be an m^6^A reader which in the mouse brain controls oligodendroglial specification and myelination in part via stabilization of the Olig2 mRNA (11). All three PRRC2 proteins are present in stress granules and PRRC2C is required for efficient stress granule formation, while PRRC2A and B were not tested (12). Although their molecular functions are not completely understood, the PRRC2 proteins appear to have clinical relevance. Mutations in PRRC2A are associated with multiple cancers, including non-Hodgkin-Lymphoma (13), lung cancer (14), ER-positive breast cancer (15) and hepatocellular carcinoma, where they promote progression and immune infiltration (16). A PRRC2B-ALK gene fusion was found in an aggressive malignant neoplasm (17), and PRRC2C was found to have a different pattern of alternative splicing in non-small lung tumors compared to healthy lung (18). PRRC2A is also associated to rheumatoid arthritis (19).

**Figure 1.**
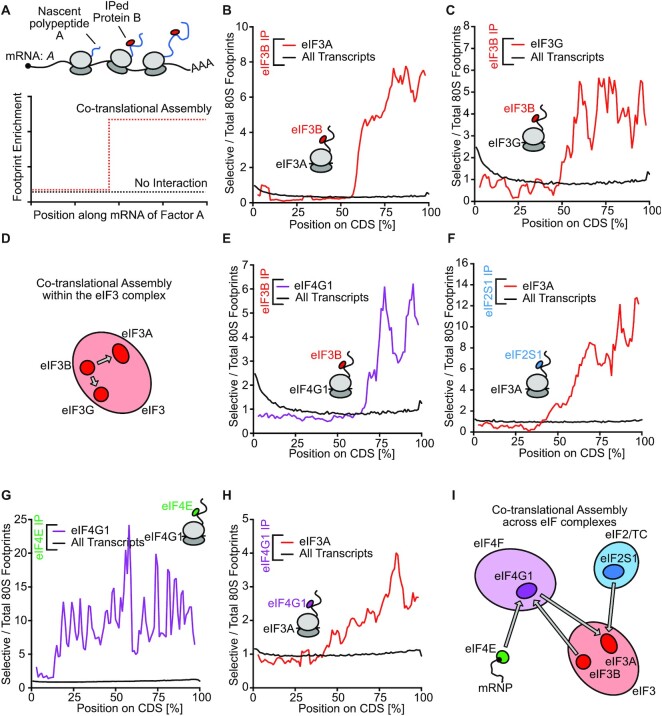
Selective ribosome footprinting reveals co-translational assembly within and across eIF complexes. (**A**) Concept of co-translational assembly and its detection by selective ribosome footprinting. In case of co-translational assembly, ribosomes bound by the bait factor B are pulled down due to interaction with the nascent polypeptide chain A. Therefore, co-translational assembly will be visible as a sudden increase in the rate of factor B bound ribosomes observed on the mRNA of gene A, with the position of this increase representing the onset of interaction between factor B and the nascent chain. (**B–D**) eIF3B interacts with nascent eIF3A (B) and eIF3G (C). Ratio of eIF3B selective 80S ribosome footprints per total 80S ribosome footprints on the eIF3A (B) or eIF3G (C) mRNA coding sequence (red) and on the main coding sequence of all other transcripts (black). Length of coding sequences is scaled between 0 and 100%. Data are an average of two biological replicates. (D) Summary of co-translational interactions within the eIF3 complex detected in eIF3B selective 80S ribosome footprinting. (**E–I**) Co-translational assembly between eIF complexes. (E) eIF3B interacts with nascent eIF4G1, (F) eIF2S1 interacts with nascent eIF3A, (G) eIF4E interacts with nascent eIF4G1 and (H) eIF4G1 interacts with nascent eIF3A. Data are an average of two biological replicates. (I) Summary of co-translational interactions between eIF complexes detected in selective 80S ribosome footprinting.

We identified the PRRC2 proteins while exploiting the concept of co-translational complex assembly. This is based on the finding that proteins can bind other proteins in a complex already when these other proteins are still incomplete, nascent polypeptides in the process of being synthesized by a ribosome. This interaction can occur as soon as the nascent polypeptide is long enough to exit the ribosome channel (Figure 1A). This occurs between nascent polypeptides and chaperones, as well as between nascent polypeptides and interacting proteins of a complex, leading to ‘co-translational chaperoning’ and ‘co-translational assembly’ of protein complexes, respectively (20–23). Co-translational assembly thereby results in a chain of physical links from the interacting protein to the nascent polypeptide, to the translating ribosome, to the mRNA coding for the nascent polypeptide. Hence co-translational assembly events can be detected when performing selective ribosome footprinting. This method is based on the sequencing of mRNA footprints that are protected by ribosomes, combined with immunoprecipitation to specifically enrich ribosomes bound to a protein of interest (21,24,25). In the case illustrated in Figure 1A, immunoprecipitation of Protein B can yield ribosome footprints on the mRNA of gene A, as soon as the interaction domain of protein A exits the ribosome channel as a nascent polypeptide (21).

In this study, we discover that PRRC2 proteins impact translation initiation of uORF-containing mRNAs by promoting leaky scanning.

## MATERIALS AND METHODS

### Cell lines and culture conditions

We cultured HeLa cells in DMEM + 10% fetal bovine serum (FBS) +100 U/ml Penicillin/Streptomycin (Gibco 15140122). Cell splitting was performed using trypsin–EDTA. HeLa cells were tested negative for mycoplasma and authenticated using SNP typing.

### Generation of knockout cell lines

PRRC2A, PRRC2B, PRRC2C and the corresponding double and triple knock-out HeLa cells were generated with the CRISPR-cas9 system. All sgRNA sequences were take from the Brunello library (26) (sequences listed in Supplementary Table S3). Oligos carrying the sgRNA sequences were cloned into pX459V2.0 via the Bpi1 site. Isogenic HeLa WT were transfected with these plasmids using Lipofectamine 2000 according to manufacturer's recommendations (Life Technologies #11668500). Twenty four hours post-transfection, cells were re-seeded into medium containing 1.5 μg/ml puromycin (Sigma-Aldrich #P9620). Three days after puromycin selection, cells were moved into normal medium (1× DMEM, 10% fetal bovine serum, 1% Penicillin/Streptomycin) and cultured to reach confluency. Single clone selection was performed by serial dilution into 96-well plates. Single clones were selected, expanded and tested for the loss of the protein of interest by immunoblotting. Double and triple knock-outs were generated by sequential knock-outs.

### siRNA mediated mRNA depletion

Cells were transfected with siRNAs using Lipofectamine RNAimax. We seeded cells at 200 000 cells per 6-well, transfected them with 3 μl of 10 μM siRNA (or siRNA Tetraplex) and 9 μl Lipofectamine reagent. Seventy two hours post transfection, cells were re-seeded for experiments. Sequences of siRNAs are provided in Supplementary Table S1.

### Ribosome footprinting

Selective 40S and 80S ribosome footprinting was carried out as published in (24,27) and as described briefly below.

### Preparation of non-denaturing cell lysates

We seeded HeLa cells at 1.5 million cells per 15 cm dish in 20 ml of growth medium 2 days before harvesting. To harvest the cells, we poured off the growth medium and washed cells quickly with ice-cold washing solution (1x PBS 10 mM MgCl_2_, 800 μM cycloheximide). After quickly pouring off the washing solution, we added freshly prepared crosslinking solution (1× PBS, 10 mM MgCl_2_, 0.025% PFA, 0.5 mM DSP, 200 μM cycloheximide) to the cells. We incubated cells with crosslinking solution for 15 minutes at room temperature with slow rocking. We then poured the crosslinking solution off and inactivated the remaining crosslinker for 5 min with ice-cold quenching solution (1× PBS, 10 mM MgCl_2_, 200 μM cycloheximide, 300 mM glycine). After removal of the quenching solution we added 150 μl of lysis buffer (0.25 M HEPES pH 7.5, 50 mM MgCl_2_, 1 M KCl, 5% NP40, 1000 μM cycloheximide) to each 15 cm dish, resulting in 750 μl of lysate. Lysis was carried out at 4°C after which we scraped cells off the dish and collected the lysate. After brief vortexing, we clarified lysates by centrifugation at 20 000 ×g for 10 min at 4°C.

### Sucrose gradient centrifugation

We collected supernatant and determined the approximate RNA concentration using a Nanodrop spectrophotometer. We added 100 U of Ambion RNAse 1 per 120 μg of measured RNA. To obtain polysome profiles, we did not add RNAse. We incubated the lysates for 5 min at 4°C and then loaded them onto 17.5–50% sucrose gradients and centrifuged them for 5 hours at 35 000 rpm in a Beckman Ultracentrifuge in a SW40 rotor. Gradients were fractionated using a Biocomp Gradient Profiler system. We collected 40S and 80S fractions for immunoprecipitation and footprint isolation. For total footprint samples, we used 40S and 80S fractions corresponding to roughly one or two 15 cm dishes. For selective footprinting samples, we used 40S and 80S fractions corresponding to roughly ten 15 cm dishes for immunoprecipitation of initiation factor bound ribosomes. For the immunoprecipitation samples we added NP40 to 1% final concentration.

### Immunoprecipitation

For immunoprecipitation, we bound antibodies to protein A or protein G magnetic dynabeads (Thermo) according to the manufacturer's instructions. For PRRC2C selective ribosome footprinting, we used 40 μl of antibody (Biomol #A303-315A) for each the 40S and 80S immunoprecipitations. We washed beads three times and then added them to the cell lysates (for protein co-IP experiments) or 40S or 80S fractions (for selective ribosome footprinting). We incubated the lysates with beads for 2 h or over-night, rotating at 4°C. Then we washed the beads three times with wash buffer (20 mM Tris pH 7.4, 10 mM MgCl_2_, 140 mM KCl, 1% NP40), including a change of reaction-vessel during the last wash. We then increased the bead volume to ∼500 μl with wash buffer. The crosslinks in total footprint fractions and IPed fractions were removed and the RNA was extracted by adding 55 μl (1/9th of volume) of crosslink-removal solution (10% SDS, 100 mM EDTA, 50 mM DTT) and 600 μl acid-phenol chloroform (Ambion) and incubating the mixture at 65°C for 45 min with shaking at 1300 rpm. We then placed the tubes on ice for 5 minutes, spun them for 5 min at 20 000 g and washed the supernatant once with acid-phenol chloroform and twice with chloroform. RNA was then precipitated with isopropanol and subjected to library preparation (see below). We used the organic phase to isolate the precipitated or total proteins. We added 300 μl of ethanol and 1.5 ml of isopropanol and incubated the solutions at –20°C for 1 h. We then sedimented the precipitated proteins by centrifugation at 20 000 g for 20 min, washed them twice with 95% ethanol 0.3 M guanidine HCl, and dried and resuspended them in 1× Laemmli buffer.

### Deep-sequencing library preparation

After RNA extraction from total and IP-purified fractions, we determined the RNA quality and integrity on an Agilent Bioanalyzer using the total RNA Nano 6000 Chip. For size selection, we ran the RNA on 15% Urea-Polyacrylamide gels (Invitrogen) and excised 25–35 nt fragments (for 80S libraries) and 20–80 nt fragments (for 40S libraries) using the Agilent small RNA ladder as a reference. We extracted RNA from the gel pieces by smashing the gels into small pieces with gel smasher tubes and extracting the RNA in 0.5 ml of 10 mM Tris pH 7 at 70°C for 10 min. We removed gel pieces by precipitating the RNA using isopropanol. We then dephosphorylated the footprints using T4 PNK (NEB) for 2 h at 37°C in PNK buffer without ATP and precipitated and purified the footprints once again using isopropanol. For 40S footprints, we depleted contaminating 18S rRNA fragments as follows. We used prevalent 18S rRNA fragments from the first round of 40S footprinting to design complementary Biotin-TEG-DNA oligonucleotides (sequences listed in (24)), ordered from Sigma-Aldrich) and mixed them in proportion to the occurrence of the fragments. We then hybridized 100 ng of RNA footprints to a mixture of these DNA oligos (in 40× molar excess) in (0.5M NaCl, 20 mM Tris pH 7.0, 1 mM EDTA, 0.05% Tween-20) by denaturing for 90 s at 95°C and then annealing by reducing the temperature by –0.1°C/s down to 37°C, followed by a 15 min incubation at 37°C. We pulled out hybridized species using Streptavidin magnetic beads (NEB) by incubating at room temperature for 15 min, and purifiying the remaining RNA by isopropanol precipitation. We then assayed footprints using an Agilent Bioanalyzer small RNA chip and Qubit smRNA kit. We used 25 ng or less of footprint RNA as input for library preparation with SMARTer smRNA-SeqKit for Illumina from Takara / Clontech Laboratories according to the manufacturer's instructions. The libaries were sequenced on an Illumina Next-Seq 550 system.

For RNA-seq libraries, we extracted total cell RNA using TRIzol and performed library preparation using the Illumina TruSeq Stranded library preparation kit. We also sequenced these RNA-seq libraries on the Illumina Next-Seq 550 system.

### Data analyses & assessment of co-translational assembly from selective 80S ribosome footprinting data

We analyzed our previously published selective 80S ribosome footprinting data (NCBI Geo GSE139391) to detect instances of co-translational assembly. For read processing and alignment we used our data analysis pipeline described in (24). For single transcript plots, we used custom scripts (available at https://github.com/aurelioteleman/Teleman-Lab) to count reads in the selective and total 80S samples for each individual transcript. Then, the ratio between selective and total 80S samples was calculated. A custom program was then used to identify co-translational assembly events, available at https://github.com/aurelioteleman/Teleman-Lab which fit the footprinting profile to a 1-step-function. This was done by selecting every position as a possible breakpoint, calculating the average of the data on each side of the breakpoint, and then calculating the sum-of-squares for distance to the average on each side of the breakpoint. The breakpoint position with the least sum of squares was selected as the optimal breakpoint for the step function. The difference in the average on the two sides of the breakpoint was then reported, yielding the ‘co-translational assembly score’.

All error bars in figures represent standard deviations. The number of biological replicates is indicated in each figure legend, as well as the statistical test used to assess significance. The significance in the change in translation efficiency upon knockdown of PRRC2A + B + C (Figure 4C) was done using Xtail analysis (28). All 80S footprint traces shown in the figures are normalized for library sequencing depth (ie the number of aligned reads in the respective library.) For 40S graphs, all graphs in all figures are normalized using the values needed to equalize the number of scanning ribosomes upstream of the metagene start codon in Supplementary Figure S4B. For Supplementary Figure S8B’, the significance of the overlap between PRRC2 target and EIF4G2 target genes was calculated using a binomial distribution.

### Preparation of cell lysates with RIPA buffer

Lysate preparation for conventional western blotting (without immunoprecipitation) was carried out as follows: Cells were seeded at 250 000 cells per 6-well. The next day, medium was removed and cells were briefly washed with DMEM. After removal of the medium, 200 μl of RIPA buffer containing 20 U benzonase, protease inhibitors and phosphatase inihiibitors were added to each well, and cells were scraped off with a pipette tip. Lysates were collected and centrifuged for 10 min at 20 000 g at 4°C. The protein concentration in the supernatant as measured by BCA. According to the protein concentration, samples were diluted to equal protein concentration and then 5× Laemmli buffer was added at 1/4th the sample volume. Samples were incubated at 95°C for 5 min and then subjected to western blotting.

### Western blotting

We ran protein samples from sucrose gradients, immunoprecipitations and lysates on SDS-PAGE gels and transferred to a nitrocellulose membrane with 0.2 μm pore size. After Ponceau staining, we incubated the membranes in 5% skim milk/PBST for 1 h, briefly rinsed them with PBST and then incubated them in primary antibody solution (5% BSA/PBST or 5% skim milk/PBST) overnight at 4°C. We then washed the membranes three times, 15 min each in PBST, incubated in secondary antibody solution (1:10 000 in 5% skim milk/PBST) for 1 h at room temperature, and then washed them again three times for 15 min. Finally, we detected chemiluminescence using ECL reagents and imaged them with a Biorad ChemiDoc imaging system. We did not strip membranes. Antibodies used for immunoblotting are listed in Supplementary Table S2.

### Cell proliferation assay

For cell proliferation assays, we carried out siRNA mediated knockdowns as described above. Seventy two hours after siRNA transduction, we re-seeded cells into 96-well plates at 1000 cells per well in 100 μl of medium, 5 wells per condition. For each day of assay, we seeded one plate. The proliferation curve was performed by harvesting the first plate on the day of re-seeding, and one plate every successive day and proliferation was assessed by Cell Titer Glo according to the manufacturer's instructions.

### Cloning

The Lamin B1 5′UTR firefly and renilla luciferase reporters and the Lamin B1 5′UTR stuORF reporter are from (29). aRaf and cRaf reporters are from (27). The reporters for different 5′UTR lengths are from (24). Renilla luciferase reporters with 5′UTRs of various genes were cloned by amplifying the 5′UTR of the gene of interest from HeLa cell cDNA and cloning it into the renilla luciferase reporter plasmid between the HindIII and Bsp119l sites. For some very short 5′UTRs, the sequence was inserted into the reporter plasmid via direct oligo cloning using the oligos indicated in Supplementary Table S4. DR1 uORF mutants were generated by site-directed mutagenesis PCR with the use of oligos listed in Supplementary Table S4, such that ATGs were mutated to TAG, near-cognate codons 1&2 into CCG and near-cognate codon 3 to GCG. The PCR products containing the mutations were cloned back into the backbone between HindIII and Bsp119I. To subclone uORF1 and uORF2 into the Lamin B1 5′ UTR, the LamB1 reporter containing a Kpn2I site from (30) was used. The DR1 region containing uORF1 and uORF2 was amplified with oligos containing Kpn2I and BshT1 sites and then cloned into the Kpn2I site of the LamB1 reporter. Reporters bearing the overlapping uORFs with different kozak strength were generated by oligo cloning between the Kpn2I and Bsp119I sites of the LamB1-Kpn2I reporter using the oligos listed in Supplementary Table S4. All constructs were verified by sequencing. All 5′UTR cloned for this manuscript are listed, including their transcript IDs and sequences, in Supplementary Table S5.

### Dual-luciferase translation reporter assay

We carried out translation reporter assays after siRNA mediated knockdowns as follows: We transfected cells with siRNAs as described above. Seventy two hours after transfection, we re-seeded cells into a 96-well plate. HeLa control cells were seeded at 8000 cells per well, and PRRC2A + B + C knockdown cells at 16 000 cells per well. We transfected cells 16–20 h after re-seeding using Lipofectamine 2000 using 100 ng of renilla luciferase plasmid and 100 ng of firefly luciferase plasmid per well. Four hours after transfection, we exchanged the medium. Luciferase assays were then performed using the Promega Dual-Luciferase assay system (cat. no. E1910) following manufacturer's instructions.

### Simultaneous detection of a uORF/oORF peptide and mainORF mNeonGreen

HEK293T-K^B^ cells (31,32), which express an MHC complex capable of displaying the SIINFEKL peptide, were transfected with siRNAs targeting either Rluc or PRRC2A + B + C. Three days after knock-down, cells were reseeded in a 12-well plate. On the following day, cells were transfected with empty vector, or with plasmids encoding mNeonGreen-PEST carrying either an oORF-less LaminB 5′UTR, or a LaminB 5′UTR with an u/oORF coding for ‘SIINFEKL’. The u/oORF has either a ‘medium’, ‘good’ or ‘strong’ start codon sequence context as shown in Figure 6E. Twenty four hours post-transfection, cells were washed with PBS, collected by trypsinization and resuspended in blocking buffer (1% BSA in 1xPBS). After 10 minutes of incubation in blocking buffer, cells were washed once more with PBS and stained for 30 minutes in the dark at 4C with 0.25 ug/ml monoclonal Antibody OVA257-264, which specifically recognizes the SIINFEKL-peptide bound to H-2Kb (Life technologies 25-5743-82). Following staining, cells were washed three times with blocking buffer, and then resuspended with 300 ul of blocking buffer and analyzed with a Guava® easyCyteTM flow cytometer running Guava Soft 3.3

### In-gradient formaldehyde crosslinking for translation initiation complex analysis

The method was carried out as described in (25). Briefly, an 80% confluent 15 cm dish of HeLa wild type cells was lysed on ice with 120 ul of lysis buffer (10 mM HEPES pH 7.5, 62.5 mM KCl, 2.5 mM MgCl 2, 1 mM DTT, 1% Triton X-100, 100 mg/ml cycloheximide, RiboLock, Protease and Phosphatase Inhibitor Cocktail). The lysate was clarified by centrifugation at 14 000 rpm for 15 min at 4°C, and loaded onto a 7–30% linear sucrose gradient with a progressively increasing concentration of formaldehyde (0.012–0.05%). The gradient was prepared with the use of a Biocomp Gradient Master, by mixing 30% sucrose (w/v) containing 0.05% formaldehyde with 7% sucrose prepared in the following buffer: 10 mM HEPES pH 7.5, 62.5 mM KCl, 2.5 mM MgCl 2, 1 mM DTT, 100 mg/ml cycloheximide. The samples were ultracentrifuged in a SW40Ti rotor at 35 000 rpm for 5 h at 4°C, followed by fractionation using a Biocomp Gradient Profiler system. Fractions 1–14 were run on an SDS-PAGE gel and analyzed for protein distribution by western blot as described above.

### Quantitative RT-PCR

Total RNA from cells with either GFP or PRRC2A + B + C knock-down was extracted using RNase Mini spin columns (Qiagen, cat. no. 74106). cDNA was generated by reverse transcription (RT) of 1ug of total RNA with random hexamer and oligo-dT + primers using Maxima H minus reverse transcriptase. The efficiency of Q-RT-PCR primer pairs was checked using a serial dilution of a sample. Quantitative RT-PCR was run on a QuantStudio3 instrument with primaQUANT SYBRGreen low ROX master mix. RNA levels were normalized to the levels of ACTB mRNA for each sample. Sequences of oligos used for Q-RT-PCR are provided in Supplemental Table S4.

## RESULTS

### Co-translational assembly occurs within and across eIF complexes

We previously published HeLa cell datasets where we immunoprecipitated ribosomes bound to translation initiation factors (eIFs) of interest, such as eIF3B or eIF4G1, and then sequenced their footprints (24). To discover novel eIF interactors, we now re-analyzed these data to search for co-translational complex assembly events, looking for footprint profiles that approximate a ‘step-function’ along the length of an mRNA, increasing suddenly as of a certain point. This makes use of the fact that footprints are detected on mRNAs encoding for interacting nascent polypeptides once the ribosome has finished translating the relevant interacting domain, causing a sudden increase in footprints on that mRNA (Figure 1A). Using an unbiased genome-wide analysis to search for such footprint profiles, we detected co-translational assembly within the eIF3 complex whereby immunoprecipitation of eIF3B yielded footprints on the mRNAs of eIF3A and eIF3G (Figure 1B-D, Supplemental Data 1). This observation is consistent with a previous report of co-translational assembly of the eIF3 complex in yeast (25). We also detect co-translational assembly between different eIF complexes where we observe co-translational interaction of eIF3B with nascent eIF4G1, of eIF2S1 (eIF2α) with nascent eIF3A, of eIF4E with nascent eIF4G1 and of eIF4G1 with nascent eIF3A (Figures 1E–H). This suggests that eIF complexes start to engage in higher order assemblies already during translation of their subunits (Figure 1E–I).

### PRRC2 proteins assemble co-translationally with eIF3 and eIF4, and are associated to preinitiation complexes

**Figure 2.**
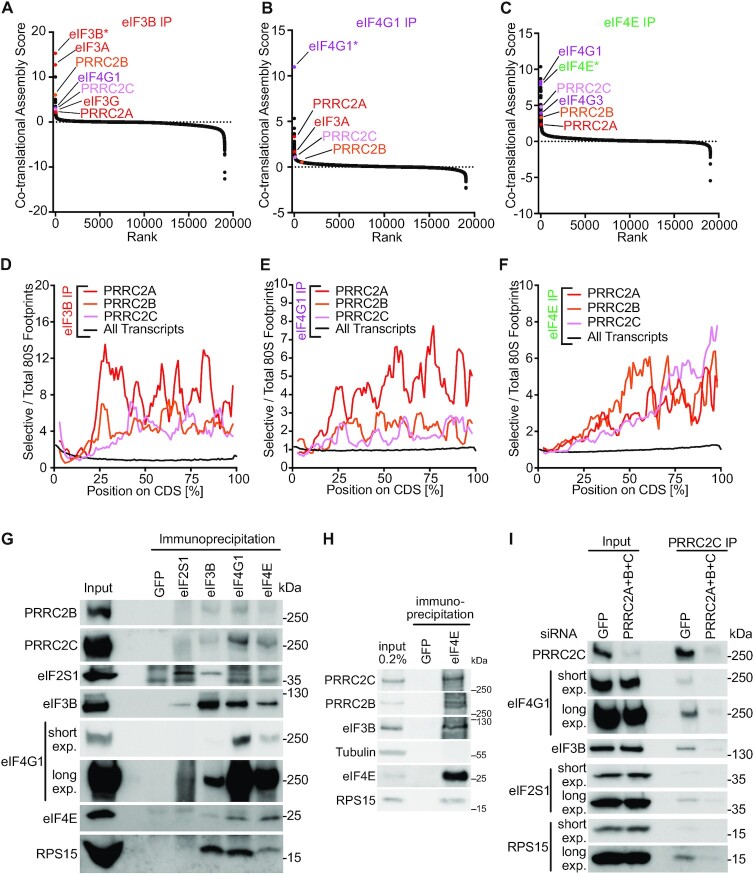
eIF3 and eIF4 interact co-translationally with PRRC2 proteins, which are associated to pre-initiation complexes. (A–C) PRRC proteins have high eIF3B (**A**), eIFG1 (**B**) and eIF4E (**C**) co-translational assembly scores. Co-translational assembly scores, reflecting how well the footprint profile on an mRNA matches a step-function that shows increased binding as of a certain point on the profile, were determined as described in Methods, for all detected genes. Values were calculated from two biological replicates. (D–F) eIF3B, eIF4G1 and eIF4E interacts with nascent PRRC2A, PRRC2B and PRRC2C. Position-resolved number of eIF3B (**D**), eIF4G1 (**E**) and eIF4E (**F**) selective 80S ribosome footprints normalized to total 80S ribosome footprints on the PRRC2A, PRRC2B and PRRC2C mRNA coding sequences and on all transcript coding sequence (black). Length of coding sequences is scaled between 0 and 100%. Data are an average of two biological replicates. (**G, H**) PRRC2B and PRRC2C co-immunoprecipitate with eIF3B, eIF4G1 and eIF4E. Immunoprecipitation of eIF3B, eIF4G1 and eIF4E, but not control IgG (anti-GFP) from whole-cell lysates of crosslinked HeLa cells co-precipitates PRRC2B and PRRC2C. Immunoprecipitations were done on cell lysates from cells crosslinked with formaldehyde and DSP as described in the Methods and (24). (**I**) Immunoprecipitation of PRRC2C co-precipitates pre-initiation complexes (PIC). Immunoprecipitation of PRRC2C from whole-cell lysates of crosslinked HeLa cells co-precipitates eIFs and ribosomal proteins. As a specificity control, siRNA mediated depletion of PRRC2A + B + C strongly reduces the amounts of co-precipitated PIC components. Immunoprecipitations were done on cell lysates from cells crosslinked with formaldehyde and DSP as described in the Materials and Methods and (24).

Genome-wide, the top proteins that interact co-translationally with eIFs were other eIFs (Figure 2A–C) (note that the protein being immunoprecipitated is often the top hit because the antibody directly immunoprecipitates the nascent polypeptide once the epitope emerges from the ribosome). In addition, amongst the top interactors were the three members of the PRRC2 family: PRRC2A, PRRC2B and PRRC2C (Figure 2A-F, Supplementary Figure S1A, B). Since these proteins have been linked to disease (13–16,19), we decided to study them in detail. We confirmed that PRRC2B and PRRC2C interact with eIFs by co-immunoprecipitation with endogenous eIF3B, eIF4G1 and eIF4E (Figure 2G, H). In the reverse setup, immunoprecipitation of PRRC2C led to co-precipitation of eIFs and of the ribosomal 40S component RPS15 (Figure 2I), suggesting it binds ribosomal preinitiation complexes. Combined knockdown of PRRC2A, PRRC2B and PRRC2C (Suppl. Figure 1C) caused the coIP signal to decrease (Figure 2I), confirming its specificity. On sucrose gradients, we observed that PRRC2B and PRRC2C sediment partly in polysomal fractions and upon RNase 1 treatment, they shift into 40S fractions (fractions 4–5, Supplementary Figure 1D), consistent with PRRC2 proteins interacting with pre-initiation complexes. Finally, a sedimentation protocol with in-gradient formaldehyde crosslinking that enables good resolution of pre-initiation complexes (33) also shows PRRC2C sedimentation in the 43–48S PIC peak (Supplementary Figure S1E). Indeed, a larger fraction of total PRRC2C protein sediments in the PIC peak compared to eIF4G1 (compare fractions 12–14 versus 2–6, Supplementary Figure S1E). Together with the fact that the copy numbers of PRRC2 proteins in HeLa cells are roughly in the same range as eukaryotic initiation factors (Supplementary Figure S1F) (34), this suggests a large fraction of initiating ribosomes are bound by PRRC2 proteins. In summary these data indicate that PRRC2 proteins are interactors of eIFs and components of the translational machinery.

### PRRC2 family proteins are required for optimal proliferation and mRNA translation

**Figure 3.**
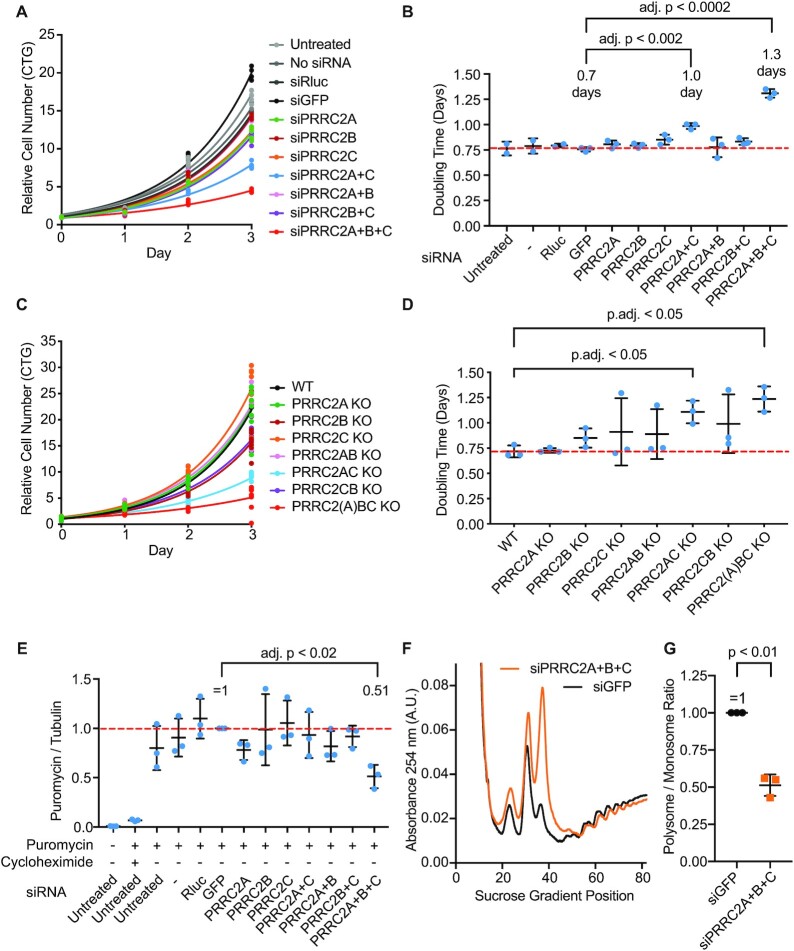
PRRC2 proteins promote proliferation and mRNA translation. (**A, B**) PRRC2 proteins are required for optimal cell proliferation. Proliferation of HeLa cells upon knockdown of PRRC2 genes was assayed using Cell titer glo (A) and doubling times were calculated by fitting the data to exponential functions (B). (A) representative experiment. siRNAs targeting Renilla luciferase (siRLuc) and GFP were used as negative controls. (B) Quantification of three biological replicates. *P*-values were calculated by multiple, two-sided t-tests, not assuming equal standard deviations and correcting for multiple testing. (**C, D**) Proliferation of HeLa cells lines knocked out for different PRRC2 proteins or combination of knockouts assayed using Cell titer glo. (C) Representative example. (D) Quantification of three biological replicates. (**E**) PRRC2 proteins are required for optimal mRNA translation. Protein synthesis in HeLa cells after PRRC2 protein depletion was assayed by Puromycin incorporation probed by western blotting. *n* = 3 biological replicates. *P*-values were calculated by multiple, two-sided *t*-tests, not assuming equal standard deviations and correcting for multiple testing. (**F, G**) PRRC2 proteins are required for optimal mRNA translation. Protein synthesis in HeLa cells after PRRC2 protein depletion was assayed by polysome profiling. (F) Representative polysome profile. (G) Quantification of the polysome/monosome ratio for three biological replicates shown. Statistical significant was assessed with paired, two-sided *t*-test, *P* = 0.0073. All error bars = std dev.

To study the function of PRRC2 proteins in translation and cell proliferation we used siRNA-mediated RNA interference to deplete these proteins from HeLa cells. Co-depletion of PRRC2A and PRRC2C led to a significant increase in the cell doubling time (Figure 3A, B, Supplementary Figure S2A–D), which was further increased upon additional depletion of PRRC2B to almost twice the doubling time of control cells (Figure 3A, B), indicating that HeLa cells require PRRC2 proteins for optimal proliferation. To confirm that this phenotype is on-target, we used two independent siRNAs targeting PRRC2C (siPRRC2C #1 and siPRRC2C #2). Both efficiently deplete PRRC2C protein in HeLa cells (Supplementary Figure 2E) and both cause an increase in cell doubling-time only when PRRC2A and PRRC2B are co-depleted (Suppl. Figure 2F). Finally, we also confirmed this phenotype using CRISPR/Cas9 mediated knockout of PRRC2A, PRRC2B and PRRC2C. Double-knockout cells lacking PRRC2A + C were significantly impaired in their proliferation (Figure 3C, D). Although we were able to generate all the double-knockout combinations, we were not able to obtain complete triple-knockout cells, suggesting that complete loss of all PRRC2 proteins is lethal. Nonetheless, we obtained one line that was fully knockout for PRRC2B/C and had mutations in all five alleles of PRRC2A, albeit mainly triplet deletions leading to loss of >3 amino acids (Supplemental Figure 3). This line (‘PRRC2(A)BC KO’) has a strong proliferation impairment similar to the triple knockdown cells (Figure 3C). Simultaneous knockdown of all three PRRC2 proteins decreases global translation rates by roughly 50%, as determined by puromycin incorporation (Figure 3E). Cells with PRRC2A + B + C knockdown also have a polysome-to-monosome ratio that is roughly half the ratio of control cells (Figure 3F, G). In summary, in the absence of PRRC2 proteins, HeLa cells have a 50% drop in translation rates and they proliferate at roughly half the rate of control cells.

We used publicly available ‘omic’ data to further investigate if PRRC2 proteins are essential. Both PRRC2B and PRRC2C whole-body knockout mice are not viable (35). PRRC2A KO mice have not been made, but PRRC2A KO in neurons causes significant impairment in neural development and survival (11). Additionally, heterozygous PRRC2C KO mice display abnormal phenotypes in behavior, vocalization, hearing and hair pattern (35). Consistent with these observations, predicted loss-of-function variants of PRRC2 genes are strongly depleted (10-fold less frequent than expected) from the general human population (36), which represents a strong signature of haploinsufficiency typical for eukaryotic translation initiation factors (eIF), ribosomal proteins (RP) and other essential genes. In summary, PRRC2 proteins are likely essential to mammalian life and subject to purifying selection to a similar degree as eIF and RP genes.

### PRRC2 family proteins are required for efficient translation of mRNAs containing uORFs

**Figure 4.**
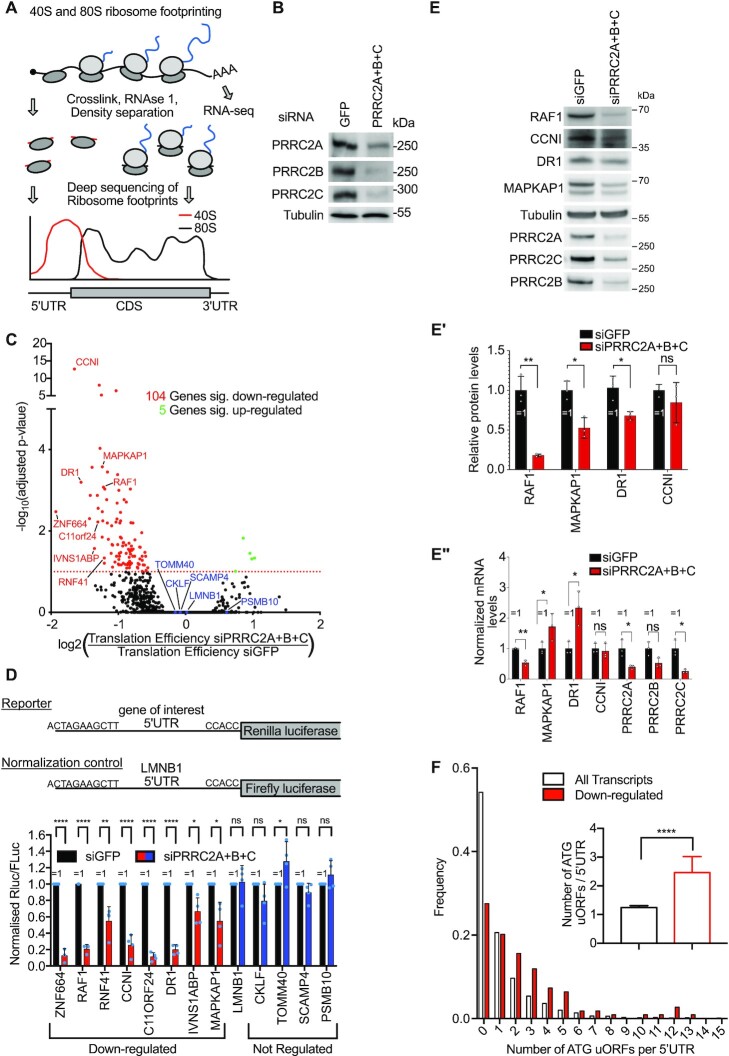
PRRC2 proteins promote translation of mRNAs containing uORFs. (**A**) Principle of 40S and 80S ribosome footprinting. Polysomes containing 40s scanning and 80S translating ribosomes are fixed in cells, RNAse treated in whole cells lysates and separated by density centrifugation. Ribosome footprints are prepared into libraries and analyzed by next-generation sequencing. (**B**) Efficiency of PRRC2 protein depletion for Ribosome footprinting experiments as determined by western blotting. Result is representative of two biological replicates. (**C**) PRRC2 proteins act as translational activators. Volcano plot showing 104 significantly down-regulated (red) and 5 significantly up-regulated (green) genes at the translational level upon PRRC2A + B + C depletion. Plot shows log_2_(fold-change in Translation Efficiency) and adjusted *P*-values for all detected genes, as obtained by Xtail analysis. (**D**) PRRC2 proteins impact translation of mRNAs in virtue of their 5′UTRs. Activity of luciferase reporters carrying the 5′UTRs of down-regulated (red) and non-target (blue) genes from (C) upon PRRC2A + B + C depletion in HeLa cells. n = 3–4 biological replicates. *P*-values calculated by unpaired, two-sided, t-test and adjusted for multiple testing. *P*-values: *Padj. <0.05, **Padj. <0.005, ****Padj. < 0.00005, ns: *P*adj. >0.05. (**E–E’’**) Combined knockdown of PRRC2A + B + C causes reduced levels of RAF1, MAPKAP1, and DR1 proteins. (E) Representative immunoblot of control (siGFP) and PRRC2 knockdown cells. (E’) Quantification of protein levels from 3 biological replicates. (E’’) Corresponding mRNA levels from control and knockdown cells show that MAPKAP and DR1 mRNA levels do not decrease in PRRC2 knockdown cells. (**F**) uORFs are enriched in mRNAs affected by PRRC2 proteins. Histogram of the frequency of ATG-initiated uORFs per 5′UTR in PRRC2 dependent (*n* = 105) and independent (*n* = 5775) genes. Insert: Average number of ATG-initiated uORFs in PRRC2 dependent (*n* = 105) and independent (*n* = 5775) genes. Inset: mean number of uORFs per transcript; error bars: 95% confidence intervals. *P*-value < 0.0001 as calculated by Mann-Whitney test. All error bars = std dev.

To study the role of PRRC2 proteins in mRNA translation, we performed 40S and 80S ribosome profiling (24), which detect footprints from initiating and elongating ribosomes, respectively (Figure 4A), in control cells or cells simultaneously depleted for all three PRRC2 proteins (Figure 4B). Note that unlike puromycin incorporation measurements or polysome profiling, ribosome footprinting normalizes away global changes in translation rates, revealing only shifts in ribosome localization within and between mRNAs. On average, transcriptome-wide, we saw no obvious changes in the distribution of 40S footprints scanning in 5′UTRs (Supplementary Figure S4A). In the absence of PRRC2A/B/C, there are 30% fewer 40S footprints aligning 25–40 nt upstream of start codons (Supplementary Figure S4B), which correspond to ribosomes directly on start codons but with large footprints due to the presence of initiation factors (24). Since this graph is normalized for the number of scanning 40S ribosomes approaching the start codon (80–100nt upstream), this potentially suggests that in the absence of PRRC2 proteins, ribosomes initiate more quickly at start codons. When we look at 80S footprints (Supplementary Figure S4D–F), we observe a mild defect in termination or recycling, seen as a 55% higher accumulation of 80S ribosomes on main ORF stop codons (arrow, Supplementary Figure 4F) as well as the expected queuing of a second 80S ribosome 50nt in front of the stop codon (arrowhead, Supplementary Figure 4F). We and others previously found a recycling defect in cells lacking DENR, a protein that promotes translation re-initiation downstream of certain uORFs (27,37). In the case of DENR, ribosome footprinting revealed an increase in 40S footprints but not 80S footprints on stop codons, indicating a defect in recycling of post-termination 40S ribosomes (27,37). Unlike for DENR, knockdown of PRRC2A + B + C causes an accumulation of 80S but not 40S footprints on stop codons (Supplementary Figure 4C), indicating that the recycling defect occurs at the level of termination or 80S splitting. Nonetheless, these global phenotypes are very mild, suggesting PRRC2 protein may rather play a role in translating specific mRNAs.

To test if there are specific mRNAs that are affected by PRRC2 depletion, we calculated translation efficiency (ribosome footprints/total mRNA) for each mRNA and found using Xtail (28) that mRNAs from 104 genes are translationally downregulated upon PRRC2 loss-of-function, whereas only 5 are significantly up-regulated (Figure 4C). Thus, PRRC2 proteins appear to promote translation, and to do so on a specific set of mRNAs. To confirm these results, we generated luciferase reporters carrying the 5′UTRs of eight predicted PRRC2 targets and four non-targets as negative controls. Indeed, simultaneous knockdown of PRRC2A + B + C caused translation of the target reporters to drop, but not of the control reporters (Figure 4D). As expected, total protein levels for targets such as RAF1, MAPKAP1 or DR1 also drop significantly upon PRRC2A + B + C knockdown (Figure 4E–E’). In contrast, mRNA levels for these targets either do not drop (MAPKAP1, DR1), or drop less strongly than the protein levels drop (RAF1) (Figure 4E’–E’’), characteristic of impaired translation. In sum, these data confirm the ribosome footprinting data, and indicate that the translation of these targets is PRRC2-dependent due at least in part to a feature in their 5′UTRs.

We noticed that PRRC2 targets have on average 60% longer 5′UTRs compared to non-target mRNAs (Supplemental Figure 5A). To test if 5′UTR length *per se* is the factor determining PRRC2 dependence, we compared two luciferase reporters with 5′UTRs of differing length (26nt versus 728nt) whereby the longer 5′UTR was generated by multimerizing the shorter 26nt sequence, so as to increase its length without introducing additional regulatory elements (24). This shows that 5′UTR length does not determine PRRC2-dependence (Supplementary Figure S5B), suggesting that PRRC2 targets carry specific features in their longer 5′UTRs.

We searched for features shared by 5′UTRs of PRRC2 targets and noticed that they are enriched for the presence of 2 or more uORFs (Figure 4F). Since this raises the possibility that uORFs cause PRRC2-dependence, we first asked if we could find a PRRC2-dependent gene that does not have uORFs in its 5′UTR. Of the 104 target genes, 16 do not have an ATG-initiated uORF. Since uORFs often start with a near-cognate start codon (38), we looked at our 80S footprinting data and found that 7 of these 16 mRNAs had clear 80S footprints in their 5′UTRs, indicating the presence of actively translating ribosomes. Hence, since we were looking for mRNAs without uORFs, we excluded these 7 mRNAs. Of the remaining 9 targets, 8 had clear 40S footprints in their 5′UTRs but not 80S footprints, identifying them as potential PRRC2 targets with no uORFs. We therefore cloned these 5′UTRs into luciferase reporters and tested their PRRC2-dependence. Unlike the luciferase reporters for the other target genes (Figure 4D), none of these dropped in expression upon PRRC2 knockdown (Supplementary Figure S5C), indicating that the selection for putative targets lacking uORFs strongly enriched for false-positives from the riboseq data. In sum, all the mRNAs whose translation we could confirm to be PRRC2-dependent, contain uORFs.

### uORFs are required for PRRC2-dependence

**Figure 5.**
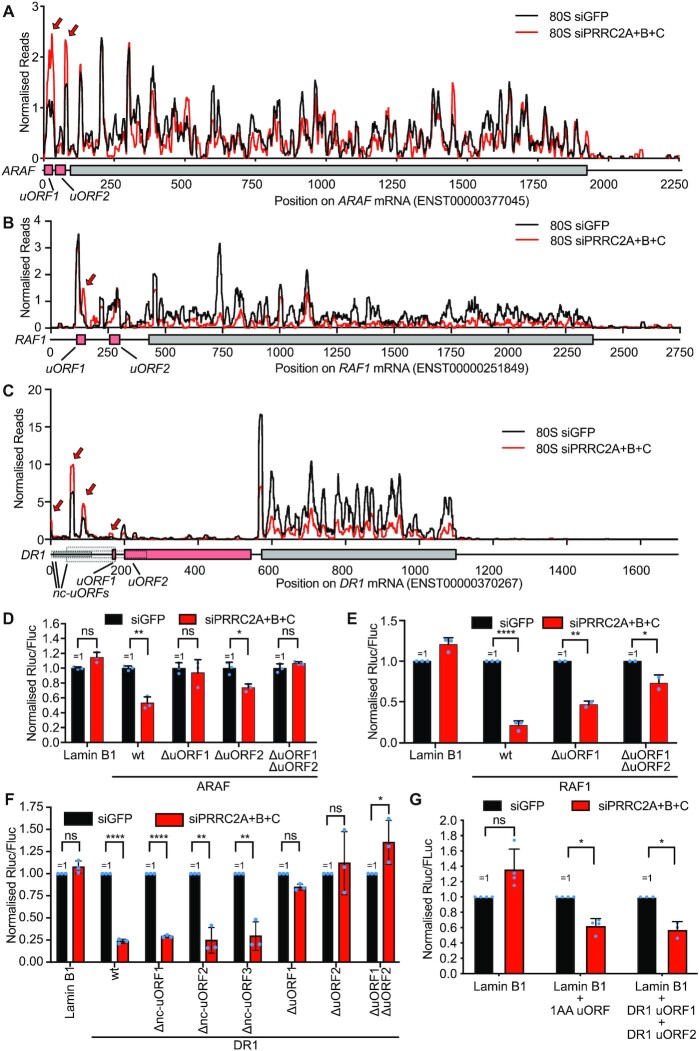
uORFs are required for PRRC2-dependence. (**A–C**) Ribosome footprints on ARAF (A), RAF1 (B) and DR1 (C) mRNAs in control (black) or PRRC2-knockdown cells (red). Read counts were normalized to sequencing depth. Graphs were smoothened with a sliding window of 11nt (A), 16 nt (B) or 10nt (C). mRNA features: uORFs (pink), main ORF (grey) and uORFs with near-cognate start codons (dotted lines) that have evidence for initiation from aggregate harringtonine traces in GWIPs-viz (66). Red arrow indicates 80S accumulation on uORF. (**D–F**) uORFs are required for PRRC2 dependence of ARAF(D), RAF1 (E) and DR1 (F) luciferase reporters. Mutation of the uORFs removes or severely blunts the PRRC2-dependence. *n* = 2–3 biological replicates (E) or three biological replicates (D, F) ± standard deviation. *P*-values calculated by unpaired, two-sided, *t*-test and adjusted for multiple testing. *P*-values: *P.adj. <0.05, **Padj. <0.005, ****Padj. <0.00005, ns: *P*adj. >0.05. (**G**) Presence of uORFs is sufficient to induce PRRC2 translational regulation. Dual-luciferase translation reporter assay of LMNB1 (negative control) 5′UTR reporter and LMNB1 reporter with insertion of 1AA uORF or DR1 uORF1 + uORF2. Schematic diagrams of the reporters are shown in Supplementary Figure S4D. *n* = 3–4 biological replicates. *P*-values calculated by unpaired, two-sided, *t*-test and adjusted for multiple testing. *P*-values: *Padj. <0.05, ns: *P*adj. >0.05. All error bars = std dev.

To test if uORFs are required for a 5′UTR to be PRRC2 dependent, we analyzed the luciferase reporters carrying 5′UTRs of 3 PRRC2-targets—ARAF, RAF1 and DR1. Both ARAF and RAF1 have 2 uORFs that are clearly translated, as can be seen from the presence of 80S footprints in these regions (Figure 5A-B). DR1 has a more complex 5′UTR with two uORFs that start with an AUG, and >7 putative uORFs that start with near-cognate start codons, some of which appear translated due to the presence of 80S footprints in these regions (Figure 5C). As expected, all three reporters carrying the wildtype 5′UTR sequence of ARAF, RAF1 or DR1 are PRRC2-dependent (‘wt’, Figure 5D–F). We confirmed that these results are due to changes in mRNA translation and not mRNA levels by normalizing the Rluc activity to *Rluc* mRNA measured by qPCR and obtained the same results (wt RAF1 reporter drops by 74%, *t*-test < 0.0001, Supplementary Figure S5D). To test if the uORFs are required, we mutated the first two nucleotides of each start codon. For all three genes, combined mutation of the two uORF ATGs abolished or strongly diminished the PRRC2-dependence (‘ ΔuORF1 ΔuORF2’, Figure 5D, E). Hence uORFs are a key element determining PRRC2-dependence. Consistent with this, introduction of a synthetic uORF composed of a start codon followed directly by a stop codon into a negative control luciferase reporter carrying the Lamin B1 5′UTR was sufficient to impart PRRC2-dependence (Figure 5G, Supplementary Figure S5E). Likewise, introduction of the two DR1 uORFs into the Lamin B1 reporter also rendered it PRRC2-dependent (Figure 5G, Supplementary Figure S5E). In sum, uORFs are required to render a 5′UTR dependent on PRRC2 for efficient translation.

### PRRC2 proteins promote translation of uORF containing mRNAs via leaky scanning

**Figure 6.**
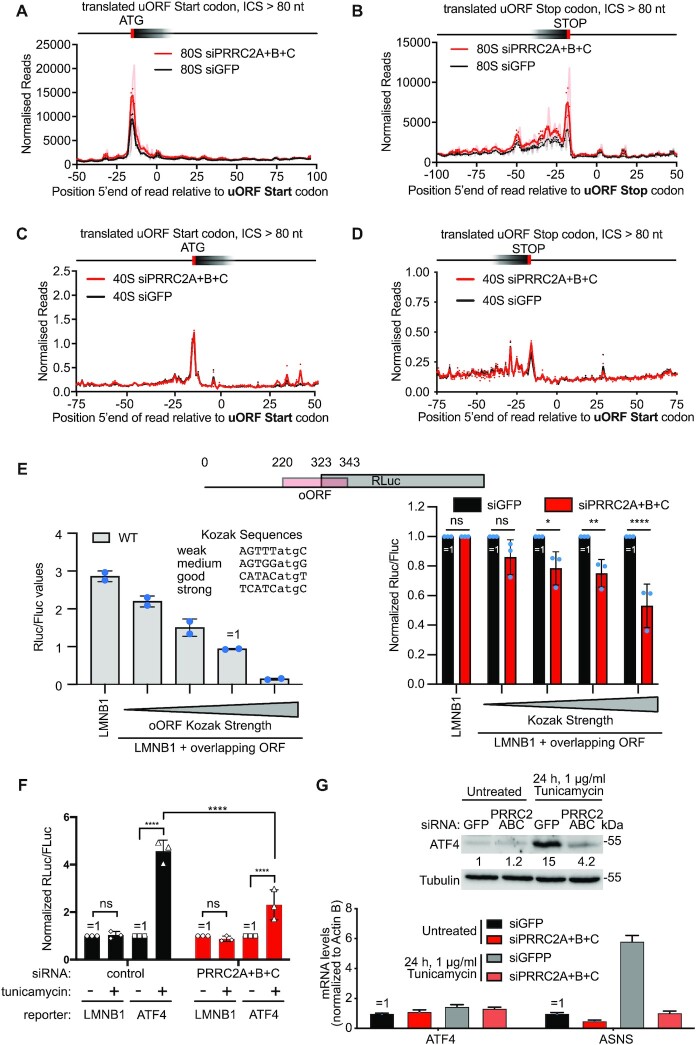
PRRC2 proteins promote leaky scanning. (**A–D**) PRRC2 knockdown causes accumulation of 80S ribosomes (A, B), but not scanning 40S ribosomes (C-D) on AUG-initiated uORFs. Metagene plots of 80S (A-B) or 40S (C-D) footprints near uORF start (A, C) or stop codons (B, D) of translated, AUG-initiated uORFs with an intercistronic space between uORF stop and mORF start codon of at least 80 nucleotides. 80S graphs are normalized to library size. Each 40S library graph is normalized by the number required to equalize the regions indicated with ‘ = 1’ (scanning ribosomes) in Supplementary Figure S4B. Solid curves show data after triplet periodicity has been removed by averaging with a sliding window of 3 nt length. Shaded curves show non-smoothed data with triplet periodicity. (**E**) PRRC2 proteins promote leaky scanning. Luciferase reporter assay of Lamin B1 5′UTR reporters containing overlapping uORFs with start codons flanked by Kozak sequences of different strengths. Left panel: RLuc/FLuc ratios for the indicated reporters in wildtype cells shows the expected result that oORFs with stronger Kozak sequences inhibit RLuc translation more strongly. Right panel: PRRC2 knockdown reduces RLuc expression, and hence leaky scanning. n = 3 biological replicates ± standard deviation. *P*-values calculated by unpaired, two-sided, *t*-test and adjusted for multiple testing. *P*-values: *Padj. <0.05, **Padj. <0.005, ****Padj. <0.00005, ns: *P*adj. >0.05. (**F**) PRRC2 proteins are required for stress-dependent ATF4 induction via leaky scanning. Cells with PRRC2A + B + C or control siRNA knock-down were transfected with luciferase reporter bearing LMNB1 (negative control) 5′UTR or ATF4 5′UTR reporter. Induction of integrated stress response with 1 ug/ml tunicamycin shows blunted induction of ATF4 reporter upon PRRC2A + B + C knock-down. *n* = 3 biological replicates ± standard deviation. *P*-values: *Padj. <0.05, **Padj. <0.005, ****Padj. <0.00005, ns: *P*adj. >0.05. (**G**) PRRC2 knockdown causes impaired induction of ATF4 protein (top panel) and impaired transcriptional induction of the ATF4 target gene ASNS in response to stress (1 μg/ml tunicamycin 24 h). All error bars = std dev.

The data presented above indicate that PRRC2 proteins somehow promote translation of a main ORF that is downstream of uORFs. There are essentially two mechanisms how ribosomes can overcome the inhibitory effect of uORFs. Either they scan past the uORF start codon in a process called ‘leaky scanning’, or they translate the uORF and then re-initiate translation downstream of the uORF (39). We noticed that upon knockdown of PRRC2A + B + C the number of 80S footprints within the uORFs of ARAF, RAF1 and DR1 mostly increase (red arrows, Figure 5A–C). This suggests that in the absence of PRRC2 proteins, uORFs are more translated. Indeed, this trend can be observed transcriptome-wide. We previously identified a low-stringency set of all ‘translated uORFs’ as those that contain at least one 80S footprint (24). Metagene plots for 80S footprints relative to the start and stop codons of these translated uORFs shows that upon loss of PRRC2 proteins more ribosomes initiate translation and more ribosomes terminate translation on uORFs compared to control cells (Figure 6A, B). Of note, genome-wide, the degree of accumulation of 80S ribosomes on uORF stop codons due to PRRC2 knockdown (Figure 6B) is similar to the degree of 80S accumulation on uORF start codons (Figure 6A) suggesting that the stop-codon accumulation reflects increased translation of uORFs and not a termination defect. These accumulations are not observed for scanning 40S footprints (Figure 6C, D) indicating that in the absence of PRRC2, a higher proportion of the scanning 40S ribosomes convert into elongating 80S ribosomes on uORFs. Together, these data suggest that the presence of PRRC2 proteins helps promote leaky scanning.

To directly assay leaky scanning, we built a luciferase reporter containing an overlapping ORF (oORF) that overlaps with the luciferase coding sequence, extending 20nt past its start codon (Figure 6E). Since ribosomes scan in a 5′-to-3′ direction, any ribosome that initiates on the oORF, and hence terminates downstream of the luciferase start codon, will not be able to translate luciferase. Thus, luciferase activity is a good readout for the number of ribosomes that (leaky) scan past the oORF start codon. The amount of leaky scanning depends on the strength of the oORF ATG sequence context. Hence, we constructed 4 oORF reporters with varying oORF ATG sequence contexts, from weak to strong as experimentally measured by (40). As expected, we observed that the stronger the oORF ATG context, the lower the signal of the luciferase mORF (Figure 6E, left panel), thereby experimentally re-validating the strengths of the sequence contexts we selected. Knockdown of the PRRC2 proteins caused the luciferase signal to drop for all oORF constructs (Figure 6E, right panel) indicating that PRRC2 proteins promote leaking scanning. A similar effect was observed with PRRC2(A)BC KO cells (Supplementary Figure S6A). The magnitude of the effect is larger when the oORF sequence context is strong, suggesting that PRRC2 proteins promote leaky scanning in particular when the sequence context is conducive to initiation. To confirm that reduced expression of the mORF coincides with increased translation of the oORF, we used a system that allows simultaneous detection of the oORF and the mORF (Supplementary Figure S6B). For the oORF, we cloned the sequence encoding the peptide SIINFEKL, which gets presented on the surface of HEK293T-K^b^ cells bound to MHC, and can be detected with an antibody (31,32). As a start codon sequence context for the oORF we used the same ‘medium’ or ‘good’ sequences as in Figure 6E. The mORF encodes mNeonGreen which can be detected simultaneously as the SIINFEKL peptide on a single-cell basis by FACS. This revealed that knockdown of PRRC2A + B + C causes an up-and-leftward shift of the FACS profile indicating a simultaneous drop in mNeonGreen (mORF) expression and increase in SIINFEKL peptide (oORF) expression (Supplementary Figure S6C).

We hypothesized that the increased leaky scanning caused by loss of PRRC2 proteins should also occur on mORFs. It is challenging, however, to test this with luciferase reporters, because it would occur on the start codons of both the RLuc test reporter and the FLuc normalization control reporter, so this effect would be normalized away. Therefore, we made use of the fact that the knockdown of PRRC2 proteins reduced leaky scanning more strongly on start codons in a strong sequence context than a weak sequence context (Figure 6E, right panel). We generated a series of reporters with differing ATG contexts on the RLuc main ORF (same sequences as in Figure 6E), and co-transfected them with an FLuc normalization control with a medium ATG context. If loss of PRRC2 proteins increases recognition of mORF start codons in a strong sequence context more than in a weak sequence context, then the RLuc/FLuc ratio should increase as the RLuc sequence context gets stronger. Indeed, as expected, a stronger RLuc mORF ATG context led to a larger increase in signal upon PRRC2 knockdown, compared to weaker sequence context (Supplementary Figure S6E), indicating that PRRC2 proteins also promote leaky scanning at mORF start codons. Furthermore, both experiments with the oORF or the mORF indicate that PRRC2 proteins preferentially decrease recognition of start codons in a strong sequence context.

On mRNAs containing both a uORF and a main ORF, the data presented above suggest PRRC2 knockdown should cause reduced leaky scanning at both the uORF and the main ORF start codons – two effects that can counteract each other in terms of mORF translation. Nonetheless, on mRNAs that show PRRC2 dependence, the effect on the uORF must dominate because we see reduced translation of the mORF upon PRRC2 knockdown (Figures 4D and 5D–G). We hypothesized that the balance of the two effects likely depends on the strength of the uORF start codon sequence context. To test this, we made use once again of the system allowing us to simultaneously detect the SIINFEKL short ORF and the mNeonGreen main ORF, this time placing the SIINFEKL coding sequence as an upstream ORF (Supplementary Figure S6D). Indeed, as expected, if the uORF start codon sequence context is strong, PRRC2 knockdown causes a simultaneous decrease in mORF expression and an increase in uORF expression (seen as a shift up and to the left, Supplementary Figure S6D).

### PRRC2 proteins are required for proper expression of ATF4

One gene whose translation is regulated via a mechanism involving differential start-codon recognition is ATF4 (41–43). ATF4 is the master transcription factor effector of the Integrated Stress Response. ATF4 translation increases in response to many different stresses and it drives expression of target genes that either help cells counteract the stress if the stress is mild, or genes that instruct cells to die if the stress is severe. Its pro-survival function causes ATF4 to be a powerful oncogene, enabling cancer cells to form tumors despite oxidative and nutrient stress (44). ATF4 has several uORFs that are translated, followed by an oORF that overlaps with the main ATF4 coding sequence. The oORF normally captures most of the ribosomes, causing ATF4 translation to be low. When the Integrated Stress Response is activated, scanning ribosomes bypass the oORF start codon and translate ATF4 instead. Hence, addition of tunicamycin to cells, which activates the Integrated Stress Response, causes an ATF4 luciferase reporter (27) to increase in expression (Figure 6F). In agreement with PRRC2 promoting ribosomes to scan past oORF start codons, induction of the ATF4 reporter is blunted upon PRRC2A + B + C knockdown (Figure 6F), analogous to the synthetic oORF reporters presented in Figure 6E. As expected,the overlapping uORF (uORF3) of ATF4 is required and sufficient to cause PRRC2-dependence, since mutation of uORF3 abolishes the PRRC2-dependence, whereas mutation of uORFs 1 and 2 but not uORF3 retains PRRC2-dependence (Supplementary Figure S6F). Correspondingly, PRRC2 knockdown leads to strongly reduced levels of ATF4 protein, but not mRNA, in response to cell stress (Figure 6G). In line with that observation, *ASNS* mRNA, a target of ATF4, is poorly induced in PRRC2A + B + C knockdown cells (Figure 6G, bottom panel). Thus, PRRC2 proteins are required for the proper induction of ATF4 upon cell stress. Since high tumor ATF4 activity strongly correlates with poor cancer prognosis (45–51), this could explain in part why PRRC2A is associated to cancer. Interestingly, the mechanism by which the oORF in ATF4 is bypassed upon stress is thought to involve a delay in recruitment of initiator tRNA after translation of uORF2, causing the scanning 40S ribosomes to be devoid of initiator tRNA when they reach the oORF start codon. In contrast, in our luciferase assays using an oORF without a preceding uORF (Figure 6E), the scanning 40S ribosomes contain initiator-tRNA. This suggests that PRRC2 proteins can promote leaky scanning by both ribosomes with a ternary complex, or ribosomes in the process of recruiting a new initiator-tRNA. One possible mechanism could be if PRRC2 proteins increase scanning speed – in the case of ATF4, this would increase the proportion of ribosomes that reach the oORF before recruiting a new initiator-tRNA, and in the case of other start codons it would reduce the chance of initiation by reducing dwell-time on the start codon. Future experiments will be needed to test this hypothesis.

Next, we tested whether PRRC2 depletion impairs leaky scanning by altering the levels of initiation factors known to be involved in this process. For instance, eIF1 (52), eIF1A (53) and eIF5 (54) control start codon selection and initiation fidelity and hence modulate the rate of leaky scanning. The levels of eIF1 and eIF5 are controlled in negative feedback loops to finetune start codon selection and leaky scanning (55–57). More recently eIF4G2 (also called DAP5/NAT1) has also been implicated in facilitating leaky scanning (58,59). Levels of eIF5 or eIF1A are unchanged in cells depleted of PRRC2 by knockdown or knockout (Supplementary Figure S7). Although eIF1 levels are elevated in PRRC2A + B + C KD cells (Supplementary Figure S7A, B), they are unchanged in PRRC2(A)+B+C KO cells (Supplementary Figure S7C, D), which also have elevated leaky scanning (Supplementary Figure S6A). Thus the impaired leaky scanning observed in PRRC2 depletion conditions does not depend on altered levels of eIFs. Since PRRC2 proteins associate physically with PICs and eIFs, this is consistent with a more direct role of PRRC2 proteins in the translation initiation process.

### PRRC2 proteins and EIF4G2 share a subset of target mRNAs

PRRC2 proteins have been reported to interact physically with the non-canonical initiation factor EIF4G2/NAT1/DAP5 (10), suggesting they may work together. Furthermore, in embryonic stem cells, nearly half of the mRNAs whose translation drops upon EIF4G2/DAP5 knockdown have uORFs (59). Hence we asked whether EIF4G2 and PRRC2 proteins share target mRNAs. Indeed, luciferase assays with reporters carrying the 5′UTRs of PRRC2 targets revealed that many, but not all of them, drop translationally upon EIF4G2 knockdown (blue bars, Supplementary Figure S8A–A’). This trend was also visible genome-wide when comparing the change in translational efficiency caused by PRRC2A + B + C knockdown which we measured in HeLa cells to the change in translation efficiencies caused by EIF4G2 knockout in HEK293T cells (from (60)) (Supplementary Figure S8B). This revealed that roughly one third of the transcripts that are PRRC2 targets are also EIF4G2 targets (blue dots), despite the data coming from two different cell lines, an overlap that is statistically significant (*P* < 10^−15^, Supplementary Figure S8B’). Interestingly, a combined knockdown of EIF4G2 and PRRC2A + B + C did not cause additive phenotypes on the reporters compared to knockdown of EIF4G2 only or PRRC2A + B + C only (yellow bars, Supplementary Figure S8A–A’). This genetic epistasis suggests EIF4G2 and PRRC2 proteins are working together in one pathway or as part of one complex.

### PRRC2C preferentially associate to ribosomes scanning and translating mRNAs with uORFs

**Figure 7.**
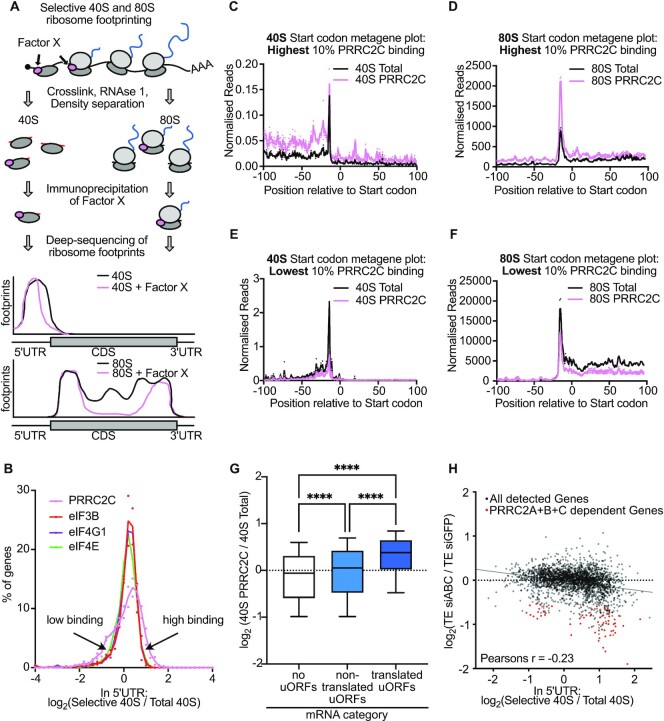
PRRC2C binding to mRNAs is heterogeneous and correlates to PRRC2-dependence. (**A**) Scheme and hypothetical example results of selective 40S and 80S ribosome footprinting. (**B**) PRRC2C binding to mRNAs is heterogenous when compared to binding of canonical eIFs. Histogram of the ratio of selective to total 40S ribosome footprints in 5′UTRs of all detected genes from PRRC2C, eIF3B, eIF4G1 and eIF4E selective 40S ribosome footprinting. *n* = 2 biological replicates. (**C–F**) Footprints of ribosomes bound to PRRC2C are elevated throughout selected mRNAs. Start codon metagene plots of mRNAs with highest (top 10%, C, D) or lowest (bottom 10%, E, F) PRRC2C binding of 40S ribosomes in 5′UTRs. High binding to 40S ribosomes in 5′UTRs (**C, E**) correlates with high binding to 80S ribosomes in 5′UTRs and coding sequences (D, F). 80S graphs are normalized to library size. Each 40S library graph is normalized by the number required to equalize the regions indicated with ‘ = 1’ (scanning ribosomes) in Supplementary Figure S4B. (**G**) Ribosomes containing PRRC2C are enriched on mRNAs containing translated uORFs. Transcripts were grouped into three categories: those without AUG-initiated uORFs (*n* = 1967), those containing bioinformatically annotated uORFs but no detectable 80S footprints on those uORFs (‘non-translated uORFs’, *n* = 2003), and those with translated uORFs (*n* = 2944). *P* values Kruskal-Wallis test followed by Dunn's multiple comparison ****Padj. <0.0001. (**H**) Genome-wide correlation between translational regulation by PRRC2 proteins and PRRC2C binding. Scatter plot of change in translational efficiency upon PRRC2A + B + C depletion versus PRRC2C binding rate to 40S ribosomes in 5′UTRs. Red dots: PRRC2-dependent transcripts from Figure 4C. Plot shows the average of two biological replicates.

We previously developed selective 40S footprinting as a method to detect when and where initiation factors bind to 40S ribosomes during the scanning and initiation process (24,25). It works by immunoprecipitating 40S ribosomes bound to the protein of interest followed by sequencing of their footprints (Figure 7A). To better understand how PRRC2 proteins function, we carried out selective 40S and 80S ribosome profiling for PRRC2C, since the PRRC2C antibody works for immunoprecipitation (Supplementary Figure S9A). As no PRRC2C can be detected in the flow-through from both 40S and 80S precipitations, we conclude that the IP efficiently depletes the samples of PRRC2C-bound ribosomes. Metagene plots of these selective ribosome footprinting data (Supplementary Figure S9B–G) revealed that on a transcriptome-wide level, PRRC2C binds 40S ribosomes evenly along 5′UTRs (Supplementary Figure S9B). 80S ribosomes tend to be more highly bound by PRRC2C in 5′UTRs compared to coding sequences and binding of PRRC2 to 80S ribosomes decreases progressively during elongation (Supplementary Figure S9E-F), as we previously observed for eIF3B, eIF4G1 and eIF4E (24). In sum, PRRC2C appears to behave similarly to standard eIFs, binding 40S ribosomes at cap recruitment and progressively dissociating from 80S ribosomes as they elongate.

To ask whether PRRC2 binds equally to all mRNAs, we calculated for each detected gene (*n* = 4707) the ratio of PRRC2C-selective 40S footprints to total 40S footprints. Interestingly, we found that PRRC2C displays heterogeneity in binding, with some mRNAs containing little PRRC2C binding and some a lot, in comparison to eIF3B, eIF4G1 and eIF4E, which bind roughly equally to all mRNAs (Figure 7B). This suggests that PRRC2C, as opposed to canonical translation initiation factors, has some specificity in its association to ribosomes on different mRNAs. To understand where this binding occurs on the mRNA, we grouped genes into two classes: the 10% with highest PRRC2 binding (*n* = 470) and the 10% with least binding (*n* = 470). mRNAs with high PRRC2 binding had high binding throughout – on scanning 40S in the 5′UTR and start codons (Figure 7C) and on elongating 80S ribosomes (Figure 7D) – whereas genes with low binding had low binding everywhere (Figure 7E, F). The same pattern could be observed on single transcripts, such as RAF1, ARAF and DR1 (Supplementary Figure 10A-C). To test whether differential binding of PRRC2C to transcripts depends on uORFs, we grouped transcripts into three categories: those without ATG-initiated uORFs, those with uORFs but no detectable 80S footprints, suggesting they are not translated, and those with translated uORFs, as described above. This revealed that ribosomes containing PRRC2C are significantly enriched on transcripts with translated uORFs (Figure 7G). Since we observe that high or low PRRC2C binding starts directly at the mRNA 5′cap (Supplementary Figure S10D, E), it is not clear how the presence of a translated uORF further downstream in the 5′UTR impacts PRRC2C recruitment mechanistically (see Discussion).

Finally, we asked whether mRNAs that have more PRRC2 binding depend more on PRRC2 for their efficient translation. Indeed, we observed a correlation between mRNAs with high PRRC2C binding and those that suffer a larger drop in translation upon knockdown of PRRC2A + B + C (Figure 7H). The drop in translation efficiency upon knockdown of PRRC2A + B + C for some mRNAs with low PRRC2 binding may be explained by PRRC2A or PRRC2B binding those mRNAs.

Thus, unlike the universal initiation factors eIF3B, eIF4G1 or eIF4E, we find that PRRC2C preferentially binds mRNAs containing translated uORFs, and that PRRC2 binding correlates with dependency on PRRC2 function for efficient translation.

## DISCUSSION

In this paper, we discover that PRRC2A, B and C impact translation initiaton. They interact with eIFs and ribosomes, and are required for the efficient translation of mRNAs containing uORFs by promoting leaky scanning. As a consequence, PRRC2 protein depletion impairs mRNA translation and cell proliferation.

To discover these regulators of translation, we employed a new rationale by using selective ribosome profiling as a tool to discover new protein interactors. This works due to the fact that the protein of interest can interact with a nascent polypeptide already during its synthesis, co-precipitating the ribosome and allowing for the sequencing of the ribosome footprint on the mRNA of the nascent polypeptide. Previously, selective ribosome footprinting was used to study the action of co-translational chaperones (20–23) and translation initiation factors (24,25). Therefore, this approach represents a new, sequencing-based method to discover protein-protein interactions that is complementary to existing methods. Particularly, the use of next-generation sequencing instead of protein mass-spectrometry may represent an advantage in terms of cost and sensitivity.

Leaky scanning is a mechanism of translational regulation first identified 30 years ago (61–63). Besides the core regulators of translation initiation (eIF1, eIF1A and eIF5), proteins influencing this process have remained largely elusive. Recently, eIF4G2/NAT1 was implicated in this process (58,59). We identify PRRC2 proteins as additional promoters of leaky scanning. PRRC2 proteins and NAT1 have been reported to interact physically (10), hence they may be working together. Indeed, in embryonic stem cells, nearly half of the mRNAs whose translation drops upon DAP5 knockdown have uORFs that are actively translated (59), fitting with our observations on PRRC2 targets. Since not all uORFs are subject to PRRC2 mediated regulation, there may be additional properties of PRRC2-dependent 5′UTRs that are yet to be uncovered (for example binding motifs in the vincinity of the uORF).

In addition to the defect in leaky scanning, we find that PRRC2 knockdown cells also have a termination or recycling defect on main ORF stop codons. Two observations suggest to us that this is likely an indirect consequence of PRRC2 knockdown, perhaps due to reduced translation of a factor involved in termination or recycling: 1) We find this defect is equally visible both on PRRC2 target mRNAs and non-target mRNAs. 2) We observe it equally on mRNAs bound highly by PRRC2C versus those bound weakly to PRRC2C. Therefore, we conclude that this is an indirect phenomenon, not directly linked to PRRC2 regulation or binding.

Previous work has linked PRRC2A to m6A modification of mRNA, describing PRRC2A as an m6A reader (11). Interestingly, m6A has also been linked to the leaky scanning mechanism regulating ATF4 induction (64). Hence it might be interesting in the future to study if there is a mechanistic link between m6A and leaky scanning. It will also be interesting in the future to study the biochemical function of PRRC2 proteins in *in vitro* translation systems, however it will require significant effort to obtain recombinant, soluble PRRC2 proteins since they are roughly 250–300 kD large.

PRRC2C displays a binding preference to a subset of mRNAs and may therefore mediate different rates of leaky scanning on different mRNAs in a cell.

It is surprising that PRRC2C-high or low binding starts directly at the cap of the mRNA, suggesting that the degree of PRRC2C binding is determined and subsequently preserved at the recruitment of the PIC to the mRNA. How this works is unclear. There may be a mechanism in *cis* (e.g. by the presence of motifs anywhere in the mRNA) or in *trans* (by the subcellular microenvironment in which the mRNA is translated) that mediates the degree of PRRC2C binding.

PRRC2 proteins are among the few known translation factors that are not conserved from yeast to human. Instead, they are conserved among vertebrates (65), yet appear to be absent for example in *Caenorhabditis elegans* and *Saccharomyces cerevisieae*. They may therefore provide a unique functionality to the translational machinery of these phyla. It is difficult to speculate why vertebrates may be more dependent on high rates of leaky scanning on subsets of their mRNAs. Answers might be provided by the generation of KO strains or by the discovery of humans with mutations in PRRC2 proteins and characterization of their phenotype.

In sum, we identify here PRRC2 proteins as regulators of translation initiation that promote leaky scanning, enabling proteins encoded by ORFs downstream of uORFs to be more efficiently translated.

## DATA AVAILABILITY

All deep sequencing datasets have been submitted to NCBI Geo (accession number GSE211440). Custom software is available on GitHub: https://github.com/aurelioteleman/Teleman-Lab.

## Supplementary Material

gkad135_Supplemental_Files
