# Supplementary material for: PRRC2 proteins impact translation initiation by promoting leaky scanning
Source: Nucleic Acids Res. 2023 Mar 3;51(7):3391–409. doi: 10.1093/nar/gkad135 (PMC10123092; doi:10.1093/nar/gkad135)
Supplement: gkad135_Supplemental_Files [file gkad135_supplemental_files.zip › Supplemental Table Legends.docx]

Supplemental Table 1: Sequences of siRNAs used in this study.

Supplemental Table 2: Antibodies used in this study.

Supplemental Table 3: Sequences of sgRNAs used for KO generation.

Supplemental Table 4: Sequences of oligos used in this study.

Supplemental Table 5: Sequences of 5’UTRs cloned into luciferase reporters.
