# Supplementary material for: PRRC2 proteins impact translation initiation by promoting leaky scanning
Source: Nucleic Acids Res. 2023 Mar 3;51(7):3391–409. doi: 10.1093/nar/gkad135 (PMC10123092; doi:10.1093/nar/gkad135)
Supplement: gkad135_Supplemental_Files [file gkad135_supplemental_files.zip › Supplemental_Table_5.docx]

| Gene | Transcript ID | 5’UTR Sequence Cloned | Manuscript Figure | Comment |
| --- | --- | --- | --- | --- |
| DR1 | NM_001938.3 | ttcctgcaaaccttccctggcatctggagggaccaccgttgccgcgtcttcggcttccacgatctgcgttcgggctacgcggccacggcggcagccactgcgactcccactgtgcctggctctgtccatattagttcccaggcggccgtcgccgttccagcagcggcagcggcagcggcagcggcggacatgttgtgaggcggcggcgcgggtgtctgaaggatggtttggccgaggcggcggcaacggcagcggggcctcgggctctatagagccgagcccgctgggtacccgcccggtaccgcggcgaggccagtgcccctggatcttgcctctgctccgacgccgttggggaccagttaggcgacagcgcccgcccctctgaggagacacgaaggtggttccccagccgctcaaatttccggaccaccgcgctttcccctcctcagcctgggctgtgctctctctagaatcctcgggcccccactttcttcccaaactcatcctaaatctctcacacacgcgagtgttcccagccctcaagccagctgctcctccgttcattttctgcaccctcttcgcaaagcaccccccgggatcactctccgagggcgactttttgagaaatctcggtggagtagtggaccagagctggggagtttttaaaagccggggcgcgagaaacaggaaggtactATG | 4D, 5C | 22 nt (tgctggcggcggcggcagcggc) of NM_001938.3 at position 271 were not present in cDNA from HeLa cells and therefore not included in the cloned sequence  Yellow: Start codons of near-cognate uORFs  Red: ATG-initiated uORFs |
| ZNF664 | NM_152437.3 | gcgctgtcccccggaggcgtctgggtgtgcggagcgcgcgcgcgcgcggctcggaggcgcacctgtgaggtgtccctgaggagagggagggcgccctgcgtccggcagaggaggcgagcatcccgctcaggtgatgaggaacccctcgcgcacccagcgcagaaggctgctgccgccggacgcctccattgtttgaccacaacaagggccggattctcacccagcaggatcctaaggcctttgtagtccttcagccactgtgggccctgcctctgcctgttcttctggaatgtcttgggggttttgatcctgtcactgtgacctgcaaatccaagagacacatctttggaagataagagagcttcttcaagaccaaaaaaggagacggcatgatacccatgtagggaattccccaaagcagggcttgccatacctggaccccgaggagcctgcttgctggaaaggctttcctgtctgatgtgcaggaggcagaatgccaaactgactcttcaaggggcaactgcaggggctcgagaccagccagcagtatctcatccttcgatacaggggatatactgtacagtcctttttctagaagtgagacatacaagattactctacaagaggaagattccaggggctcaaaaacgcaaaggtttgcactttgagagccccttggaatgttgacaactcaggatctaaaacaaagttctgtgttaatgagttacagaattcacgtggaagtcaatgtcactttataatcgataataatactgagtgaggaacactatgcaggaagaaaccttccgtagaaagacaggcagggaaaagcttaggctgaccttaaacttacctaatagagcaagcctgagatagactgccaaaatggccaaataagagactctatgaaataacagtcttgtaactgtagtaatcataaggaaattttctccttgaaatcacgataccaaatagccaccATG | 4D | 49 nt (tgggtgcgcggcgcccgcggcctggggcgctgactcccctcacttggag) of NM_001938.3 at position 271 were not present in cDNA from HeLa cells and therefore not included in the cloned sequence |
| RAF1 | NM_002880.4 | tagaatcggagagccggtggcgtcgcaggtcgggaggacgagcaccgagtcgagggctcgctcgtctgggccgcccgagagtcttaatcgcgggcgcttgggccgccatcttagatggcgggagtaagaggaaaacgattgtgaggcgggaacggctttctgctgccttttttgggccccgaaaagggtcagctggccgggctttggggcgcgtgccctgaggcgcggagcgcgtttgctacgatgcgggggctgctcggggctccgtcccctgggctggggacgcgccgaATGTGAccgcctcccgctccctcacccgccgcggggaggaggagcgggcgagaagctgccgccgaacgacaggacgttggggcggcctggctccctcaggtttaagaattgtttaagctgccaccATG | 4D, 5B | Red: ATG-initiated uORFs |
| RNF41 | NM_005785.4 | tgtctgaggcggcgacgagtggtaggagtcttctttcccctccttcccctttcccttcccttcccgggagaggctgggacccggcaccagggcagtactgtggccgctgcggcctcagctccgactgggtcaggttgcggagactccaggccgcttccagggcgagtactcctgattgtgacatcacattcatcccctgggcgatggagcttgtcactgggaaggaatactcagtcggagaatagccaacaagatgggttactgggagaatctcttcagtggcactgagtggaggcatcagggggttggagccttgtgaacagggaacctgccccccaacacttggaaggacctgggtttcagtgatccaccATG | 4D |  |
| CCNI | NM_006835.3 | tctccctccccagccttccccgcgagcggacgcggcagcgcctctgtctcgctttttcttatttttcccccctttcccctttctttttttttttttcttttcttttctcccctccccccctttcaccatttcccctcggaggcgctttccccgggcaggggcagagccggtctcaccccccgcctctccccggcccccgccgccctatggcgagagggagccccctcccaacccgggctcgagcggcggcggcctcaggccgggggtcatcatggaactaattcgctgaccgacccagcggccgcagccgtgcgtcccgctcgagcgccagcgcccgcgcccgcgccccccgatccgcttcccctttctccctcctcagttggccgagtcgtcccgcgcgcaccgcctccgcgcgcctatgagaatgaggtggtaacgggcccccggatgaccccgcgtcaccactgtgaggcctacagctctgccggggaggaggaggaggaggaagaggaggagaaggtagctacagcaagctgggtagcaggcagatccaaaggccaccATG | 4D |  |
| C11orf24 | NM_022338.4 | tatttccggcgtttgggcggggcccgggcagggcgcgctgcccggagctgcctgggttgcgctgccggccacgtccccgcgccgggcctcaggctccttcctactgtccgagggccaccaggccgccgggggcctgctgcgcccggatgcgtctgttactagagtggagagtctaccttcgtctcacatgtgccacaaaggatggcatggcccgggagtgccccaccacgtggctttcaccccctgcaaagccagacttcgcccagcgacacagtgtcaagcccacagctctccaaggaggaagatggtccaggctgggagcatccccttagcagcagcctctgatcccttggccaagcaggagggaaccattagcagcctgaggagctggctggctgggagcctcggggaccgcccagccttgctcccagctcacccccaccATG | 4D |  |
| IVNS1ABP | NM_006469.5 | tagtgtctcccggtcgcgcgtggaggtcggtcgctcagagctgctgggcgcagtttctccgcctgctgcttcggcgcggctgtatcggcgagcgagcgagttcccgcgagttctcggtggcgctcccccttcctttcagtctccacggactggcccctcgtccttctacttgaccgctcccgtcttccgccgccttctggcgctttccgttgggccgattcccgcccgcttcctcctgcttcccatcgaagctctagaaatgaatgtttccatctcttcagagatgaaccagattatgatgcatcattatcacagaagaaattcgtgtctatagcttttaaggacttgattacatcattttcaagcctgatagttttggaatcaccattagagcttaagacacacctgccttcatttcaaccacctgtcttcataccctgacgaagtgcaccttttaacactcctttgtccttggattacttaagagttcccagaaatacatttgccaccaacagagtagccaaatttataagccaccATG | 4D |  |
| MAPKAP1 | NM_001006617.3 | tgtcgtgtgcggctcggggtaatagggctgctgctcggccggccggcggcggcgagcagcaggggcatgagggctaacccgggaagcggcagctgagcgggccgggaggagcgccggtccccgtggatcccgagagtgcagagctcggggcaggggccgggaggcgtgggggagccgggccctcccctcaggaacgtgtcccggggccgacccggcccgtagtgtggaagcagcttcaggtaggtgagctcgtgaaacaatatgaagaggagaaaatagccttttaaggaaattggcccacagccaccATG | 4D |  |
| CKLF | NM_016951.4 | tatgcgcgcaagagagcgggaagccgagctgggcgagaagtaggggagggcggtgctccgccgcggtggcggttgctatcgcttcgcagaacctactcaggcagccagctgagaagagttgagggaaagtgctgctgctgggtctgcagccaccATG | 4D |  |
| TOMM40 | NM_001128917.2 | tcggttgcgcgtggcgcacggggtgggagcggagcccaggccgggagcaggcgccgccgccagtgagaaccggggccggagccgggtgcggatttgctggggctgagtcgggggcgcgcgggccctgacctctgccctctgacctctcccctagcaggccaccATG | 4D |  |
| SCAMP4 | NM_079834.4 | taagacttggcgaagcgctgcgctcgcgcccggatccctcaggcggctgcaggcttcagcctgcgctggttggtgaaaccaccATG | 4D |  |
| PSMB10 | NM_002801.4 | tagacgtgaagcctagcagaggactttttagctgctcactggccccgcttgtctggccgactcatccgcccgcgacccctaatcccctctgcctgccccaccATG | 4D |  |
| ARAF | NM_001654.5 | taagagaggcccaagatggagacggcggcggctgtagcggcgtgacaggagccccatggcacctgcccagccccacctcagcccatcttgacaaaatctaagccaccATG | 5A |  |
