# Supplementary material for: PRRC2 proteins impact translation initiation by promoting leaky scanning
Source: Nucleic Acids Res. 2023 Mar 3;51(7):3391–409. doi: 10.1093/nar/gkad135 (PMC10123092; doi:10.1093/nar/gkad135)

Supplementary Material

PRRC2 proteins impact translation initiation by promoting leaky scanning

Bohlen, Roiuk et al.

Contents

1. Legends to Supplementary Figures.. p. 1

2. Supplementary Figures .... p. 10

Legends to Supplementary Figures

Supplemental Figure 1: Co-translational interactions within and across eIF complexes.

(A) PRRC proteins have intermediate to high co-translational assembly scores for interacting with eIF2S1. Co-translational assembly scores were determined as described in Methods for all detected genes. n=2 biological replicates.

(B) eIF2S1 may interact with nascent PRRC2A, PRRC2B and PRRC2C. Ratio of eIF2S1 selective 80S ribosome footprints versus total 80S ribosome footprints on the PRRC2A, PRRC2B and PRRC2C mRNA coding sequences (colored) and on the main ORF of all other transcripts (black). Length of coding sequences is scaled between 0 and 100%. n=2 biological replicates.

(C) PRRC2 protein depletion by siRNA transfection is efficient and specific. Knockdown efficiency and specificity of PRRC2 antibodies on lysates from HeLa cells treated with siRNA pools targeting the indicated genes.

(D) PRRC2 proteins migrate with ribosomal and polysomal complexes in sucrose gradients. Polysome profiling and subsequent western blotting of gradient fractions from HeLa cells in the presence and absence of RNAse 1. RNAse 1 digestion causes polysomal PRRC2 proteins to run in 40S fractions 4-5.

(E) Sucrose fractionation of translation initiation complexes. PRRC2C is detected in a free form (fractions 4-6) and co-sedimenting with translation initiation complexes (fractions12-14).

(F) PRRC2 protein copy number in HeLa cells is roughly in the same range as the copy numbers of eukaryotic initiation factors (eIF). Data from (57) were re-analyzed. Each dot is one detected protein. Proteins were grouped into those of the large ribosomal subunit (“RpL”), small ribosomal subunit (“RpS”), initiation factors (“eIF”) or PRRC2 proteins.

Supplemental Figure 2: Knockdown of PRRC2 proteins causes reduced proliferation.

(A) Immunoblot control of knockdown efficiency for the proliferation curves shown in main Figure 3A.

(B-D) PRRC2 knockdowns are efficient and reproducible. Quantifications of PRRC2A **(B)**, PRRC2B **(C)**, and PRRC2C **(D)** knockdown efficiency in the

indicated conditions as determined by western blotting. n=4 biological replicates.

(E) Two independent siRNAs targeting PRRC2C efficiently knock down PRRC2C expression. Knockdown of PRRC2C with siRNA-1 and siRNA-2 in the presence or absence of PRRC2A and PRRC2B knockdown using siRNA pools, assayed by immunoblotting. n=3 biological replicates.

(F) PRRC2C is required for optimal cell proliferation in the absence of PRRC2A and PRRC2B. Proliferation of HeLa cells after PRRC2 protein depletion was assayed using Cell Titer Glo and doubling times were calculated by fitting the data to exponential functions. n=3 biological replicates. P-values were calculated by multiple, two-sided t-tests, not assuming equal standard deviations and correcting for multiple testing.

All error bars = std dev.

Supplemental Figure 3: Molecular characterization of the PRRC2(A)BC knockout cell line.

(A) Both PRRC2B and C have mutations that cause frameshifts, resulting in truncated proteins, for all alleles detected. For PRRC2A, one allele has a frameshift mutation, whereas 4 alleles all have triplet deletions, leading to loss of >3 amino acids.

(B-B') Immunoblot analysis of PRRC2A, PRRC2B and PRRC2C protein levels in the various PRRC2 knockout cell lines. The PRRC2C antibody is weak, and detects a background band similar in size to PRRC2C (B) which can be size-

resolved if the gel is run extensively (B'). The specific PRRC2C band is indicated with an arrowhead.

Supplemental Figure 4: 40S and 80S ribosome footprinting upon PRRC2 depletion.

(A-F) Metagene plots of 40S **(A-C)** and 80S **(D-F)** ribosome footprints in control (black) or PRRC2 knockdown cells (red). Ribosome footprints were counted on all detected transcripts relative to the mRNA 5'UTR **(A, D,** scaled to 5'UTR length in %**)**, main ORF start codon **(B, E)** or main ORF stop codons **(C, F)**. Solid curves in **(D-F)** show data after triplet periodicity has been removed by averaging with a sliding window of 3 nt length. Shaded curves show data without smoothing where triplet periodicity is visible. 80S graphs are normalized to library size. Each 40S library graph is normalized by the number required to equalize the regions indicated with "=1" (scanning ribosomes) in panel B. These normalization values are then applied to all ribosome footprinting graphs in the manuscript.

Supplemental Figure 5: Presence of uORFs is required and sufficient for translational regulation by PRRC2 proteins via leaky scanning.

(A) 5'UTR length of PRRC2 targets (as determined by X-tails analysis) and non-targets. Whiskers indicate top and bottom deciles while boxes indicate

quartiles, the bar in the box represents the median. p-value < 0.0001 as calculated by Mann-Whitney test.

(B) 5'UTR length does not affect PRRC2 dependence. Dual-luciferase translation reporter assay of RLuc reporters carrying the LMNB1 5'UTR (negative control), or a synthetic 5'UTR length composed of a 26nt sequence lacking uORFs, either singly or multimerized. Reporter activity assessed upon control or PRRC2A+B+C knockdown. n=2 biological replicates.

(C) 5'UTRs lacking uORFs do not impart PRRC2 dependence to luciferase reporters. Dual-luciferase translation reporter assay of LMNB1 (negative control), DR1 (positive control) and uORF-less potential PRRC2 target 5'UTRs assessed upon control or PRRC2A+B+C knockdown. n=3 biological replicates.

(D) Confirmation of translational regulation of the RAF1 5'UTR by PRRC2 proteins. The Rluc translation reporter signal for each 5'UTR construct was normalized to the respective Rluc mRNA levels determined by qPCR. P-values were calculated by unpaired, two-sided, t-test and adjusted for multiple testing. n=7 biological replicates.

(E) Schematic diagram of the luciferase reporters used in main Figure 5G.

Error bars in B-D = std dev.

Supplemental Figure 6: PRRC2 promotes leaky scanning

(A) PRRC2 proteins promote leaky scanning. Dual-luciferase translation reporter assay of a LMNB1 5'UTR reporter (negative control) and the same LMNB1 reporter with insertions of overlapping uORFs with start codons flanked

by Kozak sequences of different strengths (as shown in main Fig. 6E). Activity assessed in control cells or PRRC2(A)BC KO cells. n=3 biological replicates.

(B) Schematic diagram illustrating the setup for detecting simultaneously a small peptide produced by a uORF or oORF and fluorescent mNeonGreen encoded by the main ORF. The short peptide SIINFEKL is presented on the cell surface by MHC-I and detected using a monoclonal antibody.

(C) PRRC2 knockdown causes reduced expression of the mORF and increased expression of the oORF, seen as a shift in the cell population up and to the left, when the sequence context of the oORF start codon is strong (same sequences used as in main Fig. 6E).

(D) PRRC2 knockdown causes reduced expression of the mORF and increased expression of the uORF, seen as a shift in the cell population up and to the left, when the sequence context of the uORF start codon is strong (same sequences used as in main Fig. 6E).

(E) Depletion of PRRC2 proteins causes increased leaky scanning also on the main ORF of Rluc. Dual-luciferase translation reporter assay on RLuc reporters with the LMNB1 (control) 5’UTR and start codon sequence contexts of different strengths (as shown in main Fig. 6E), normalized to an Fluc control reporter with the same 5’UTR (LMNB1). Activity assessed in control cells or PRRC2(A)BC KD cells. n=3 biological replicates.

(F) The overlapping uORF3 of ATF4 is required and sufficient to cause a drop in expression of the ATF4-luciferase reporter upon PRRC2 knockdown. n=3 biological replicates.

All error bars = std dev.

Supplemental Figure 7: eIF levels in PRRC2 knockdown and knockout cells

HeLa cells depleted of PRRC2 proteins by the siRNA mediated (A-B) or sgRNA (C-D) were stimulated with tunicamycin for 16 hours and then harvested for western blotting. (A-B) PRRC2-protein depletion by siRNA mediated knockdown causes an increase in eIF1 and impaired induction of ATF4. (A) Representative western blot of three biological replicates quantified in (B). (C-D) PRRC2-protein depletion by CRISPR/Cas9 mediated knockout does not perturb eIF protein levels, but impairs ATF4 induction. (C) Representative western blot of three biological replicates quantified in (D). n=3 biological replicates.

All error bars = std dev.

Supplemental Figure 8: PRRC2 proteins and EIF4G2 share common target mRNAs

(A-A') Most luciferase reporters carrying 5'UTRs of PRRC2 target genes also drop in expression upon EIF4G2 knockdown (blue bars, A), and do not show an additive drop upon combined EIF4G2 knockdown and knockdown of PRRC2A+B+C (yellow bars). (A') Immunoblot control for knockdown efficiencies.

(B-B') Many PRRC2 target mRNAs genome-wide also require EIF4G2 for efficient translation. (B) Log2-transformed fold change in mRNA translation efficiency (ribosome footprint counts / total mRNA counts) upon combined

knockdown of PRRC2A+B+C (x-axis) in HeLa cells from this study, versus log2-transformed fold change in mRNA translation efficiency upon EIF4G2 knockdown in HEK293 cells previously published in (55) (y-axis). (B') The overlap in PRRC2-target mRNAs and EIF4G2 target mRNAs is highly significant ($<10^{-15}$ calculated using a binomial distribution).

All error bars = std dev.

Supplemental Figure 9: Quality controls for PRRC2C selective ribosome footprinting

(A) Western blot of input material, IPed material, and flow-through for control (siGFP) cells, or siPRRC2A+B+C knockdown cells, used for total or PRRC2C-selective ribosome footprinting. Representative of two biological replicates.

(B-G) Metagene plots of total and PRRC2C selective 40S **(B-D)** and 80S **(E-G)** ribosome footprinting. Ribosome footprints were counted on all detected transcripts relative to mRNA 5'UTR **(B, E,** scaled to 5'UTR length in %**)**, main ORF start codon **(C, F)** or main ORF stop codons **(D, G)**. Solid curves in **(E-G)** show data after triplet periodicity has been removed by averaging with a sliding window of 3 nt length. Shaded curves show data without smoothing, where triplet periodicity is visible. 80S graphs are normalized to library size. Each 40S library graph is normalized by the number required to equalize the regions indicated with "=1" (scanning ribosomes) in Suppl. Fig. 4B.

Supplemental Figure 10: PRRC2C-selective ribosome footprints on target-gene mRNAs

(A) PRRC2C binding to 80S ribosomes on ARAF mRNA. Single transcript trace of total (black) and PRRC2C selective (pink) 80S ribosome footprints. Read counts were normalized to sequencing depth. Graphs were smoothened with a sliding window of 16 nt. mRNA features: uORFs (orange), mRNA (grey). Red arrow indicates 80S accumulation on uORF.

(B) PRRC2C binding to 80S ribosomes on RAF1 mRNA. Single transcript trace of total (black) and PRRC2C selective (pink) 80S ribosome footprints. Read counts were normalized to sequencing depth. Graphs were smoothened with a sliding window of 16 nt. mRNA features: uORFs (orange), mRNA (grey). Red arrows indicate 80S accumulation on uORFs.

(C) PRRC2C binding to 80S ribosomes on DR1 mRNA. Single transcript trace of total (black) and PRRC2C selective (pink) 80S ribosome footprints. Read counts were normalized to sequencing depth. Graphs were smoothened with a sliding window of 16 nt. mRNA features: uORFs (orange), mRNA (grey). Red arrows indicate 80S accumulation in the 5'UTR.

(D-E) 5'Cap metagene plot of the 10% lowest (D) and highest (C) PRRC2C bound mRNAs. 40S Ribosome footprints were counted on all transcripts of the respective group relative to mRNA 5'Cap.

80S graphs are normalized to library size. Each 40S library graph is normalized by the number required to equalize the regions indicated with "=1" (scanning ribosomes) in Suppl. Fig. 4B.

Suppl. Figure 1

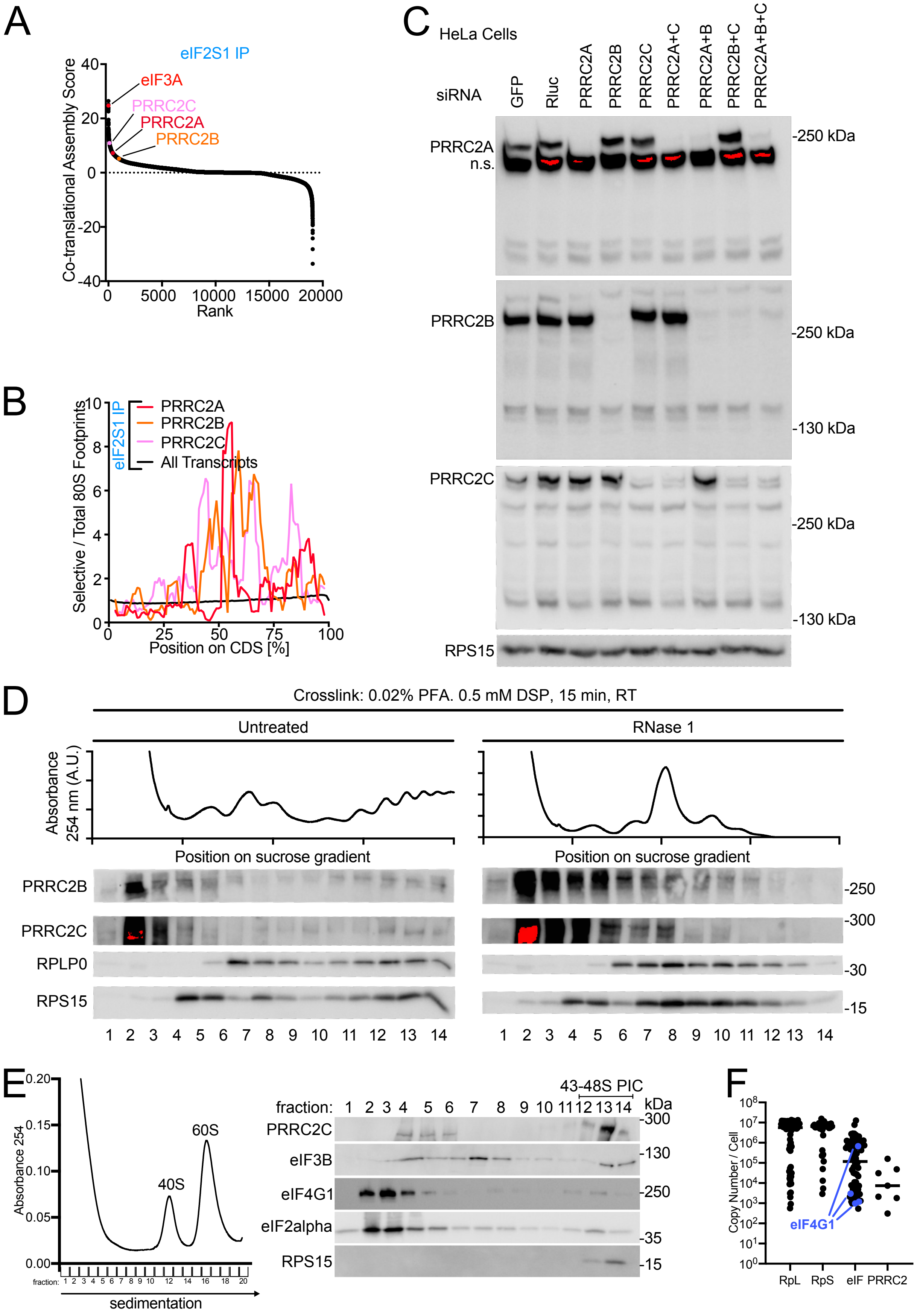

Suppl. Figure 2

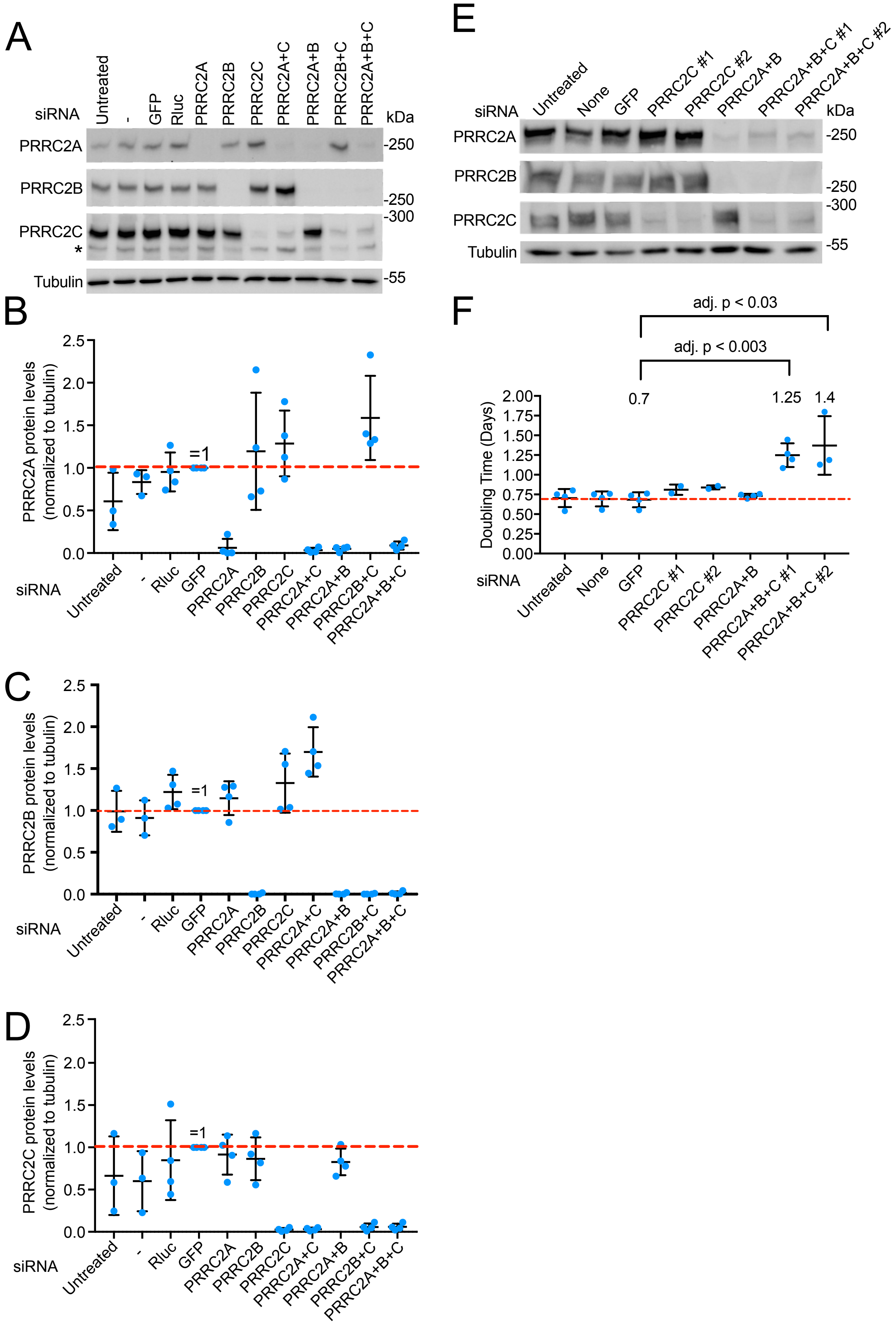

Suppl. Figure 3

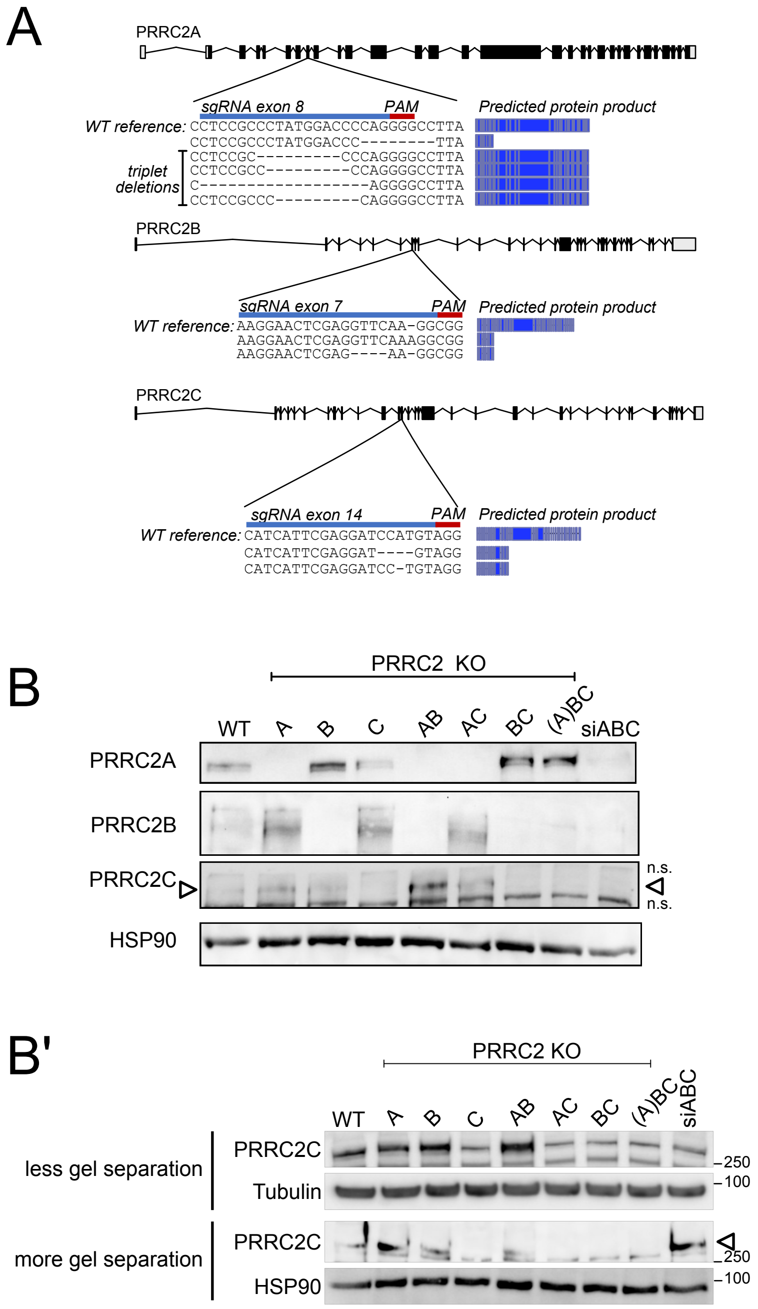

Suppl. Figure 4

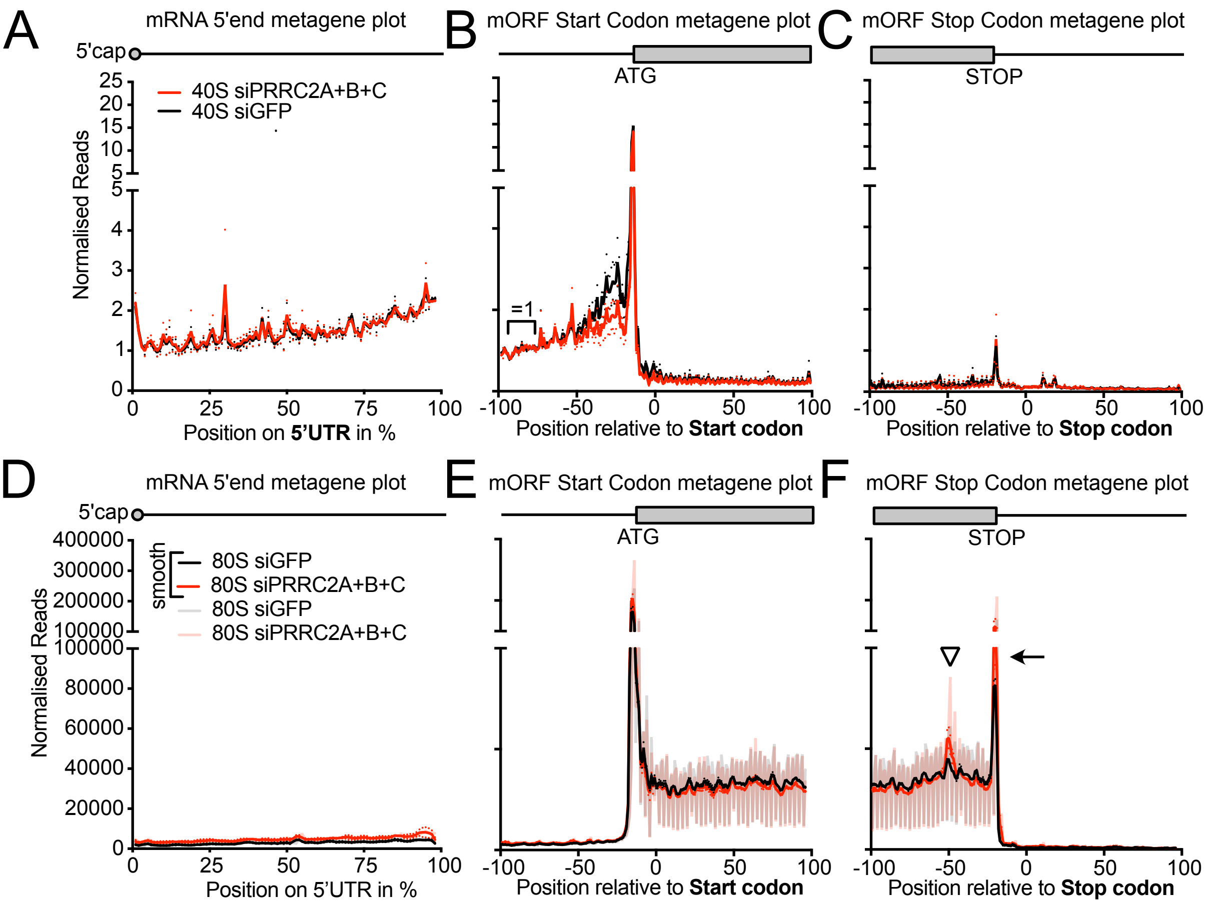

Suppl. Figure 5

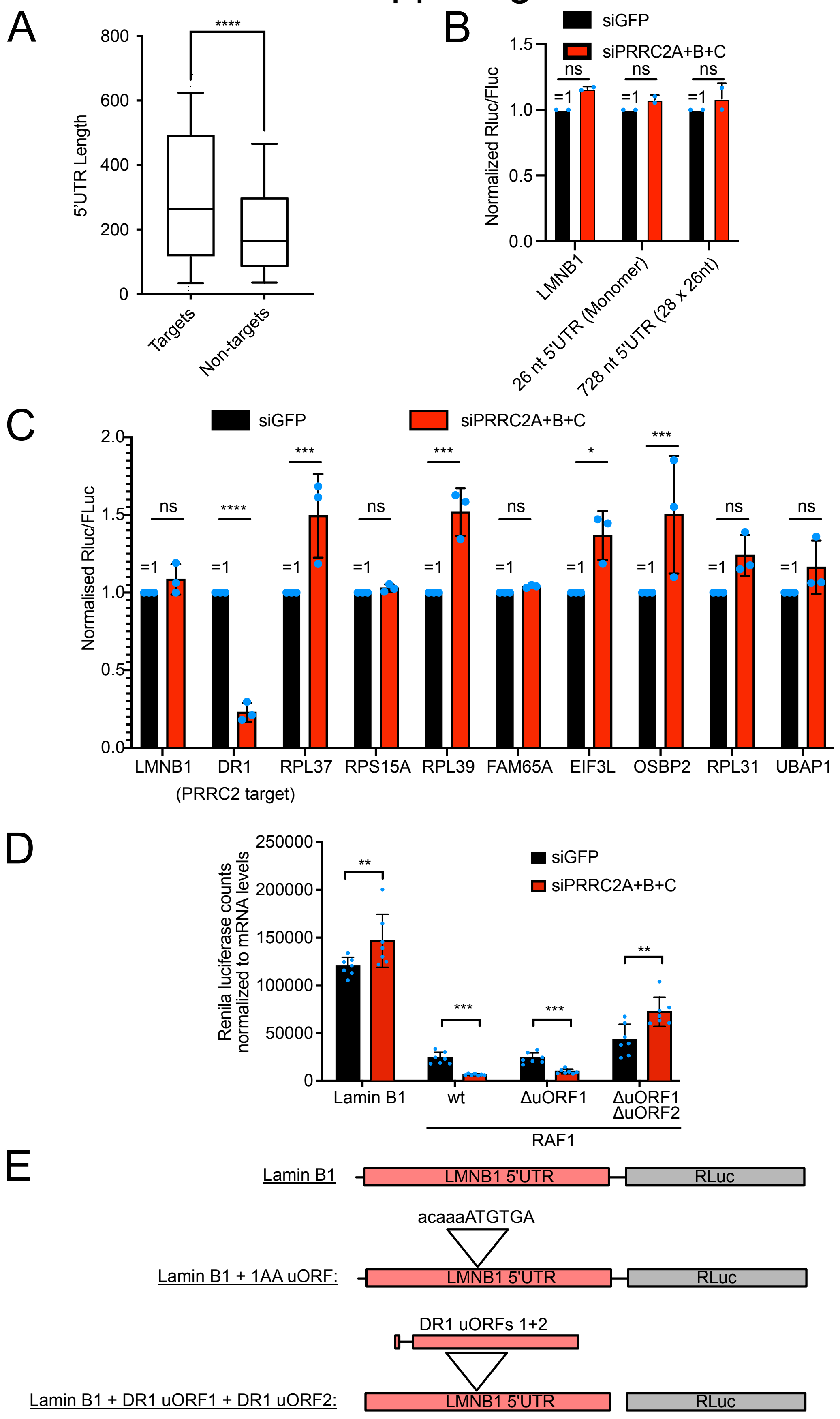

Suppl. Figure 6

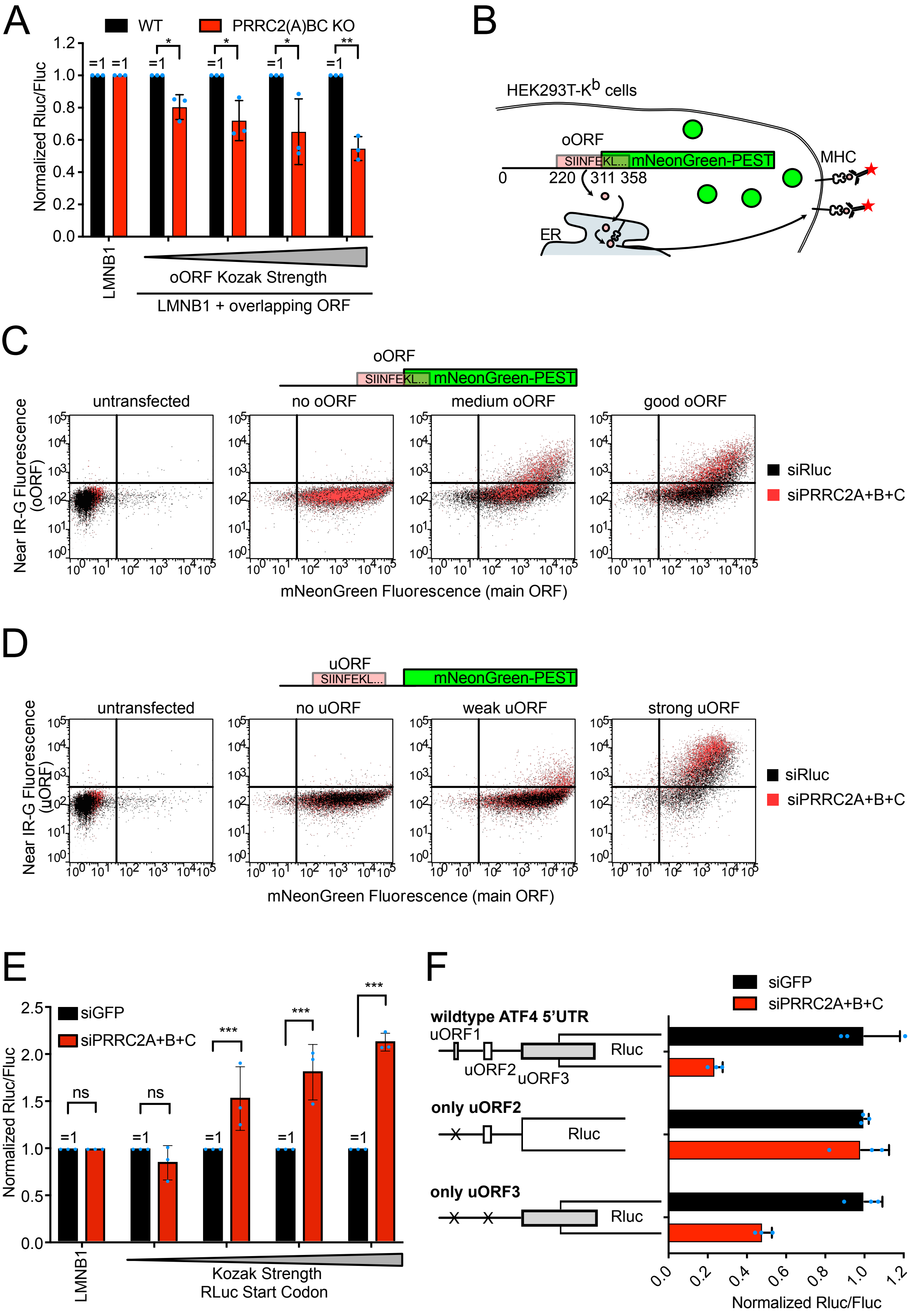

Suppl. Figure 7

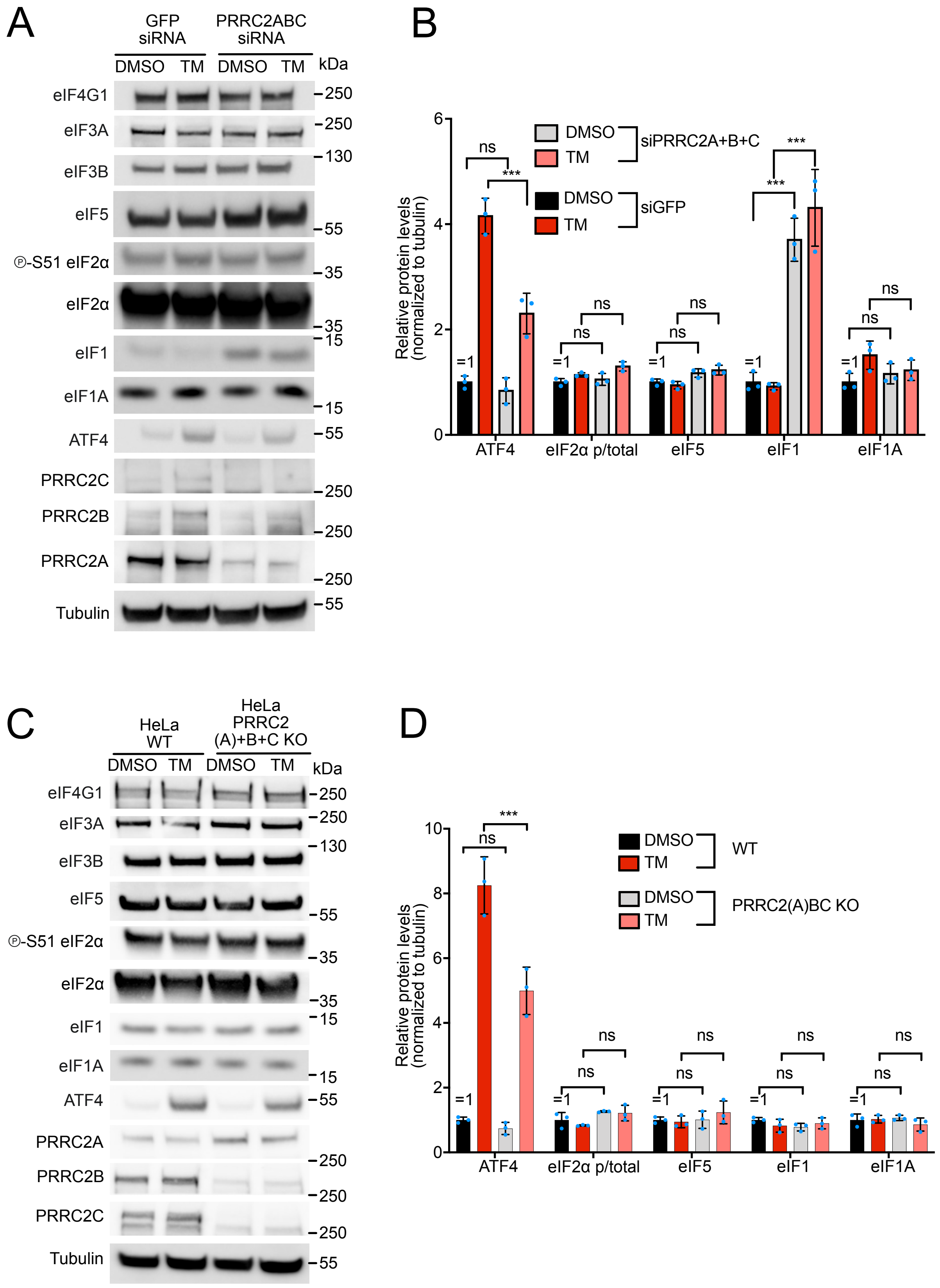

Suppl. Figure 8

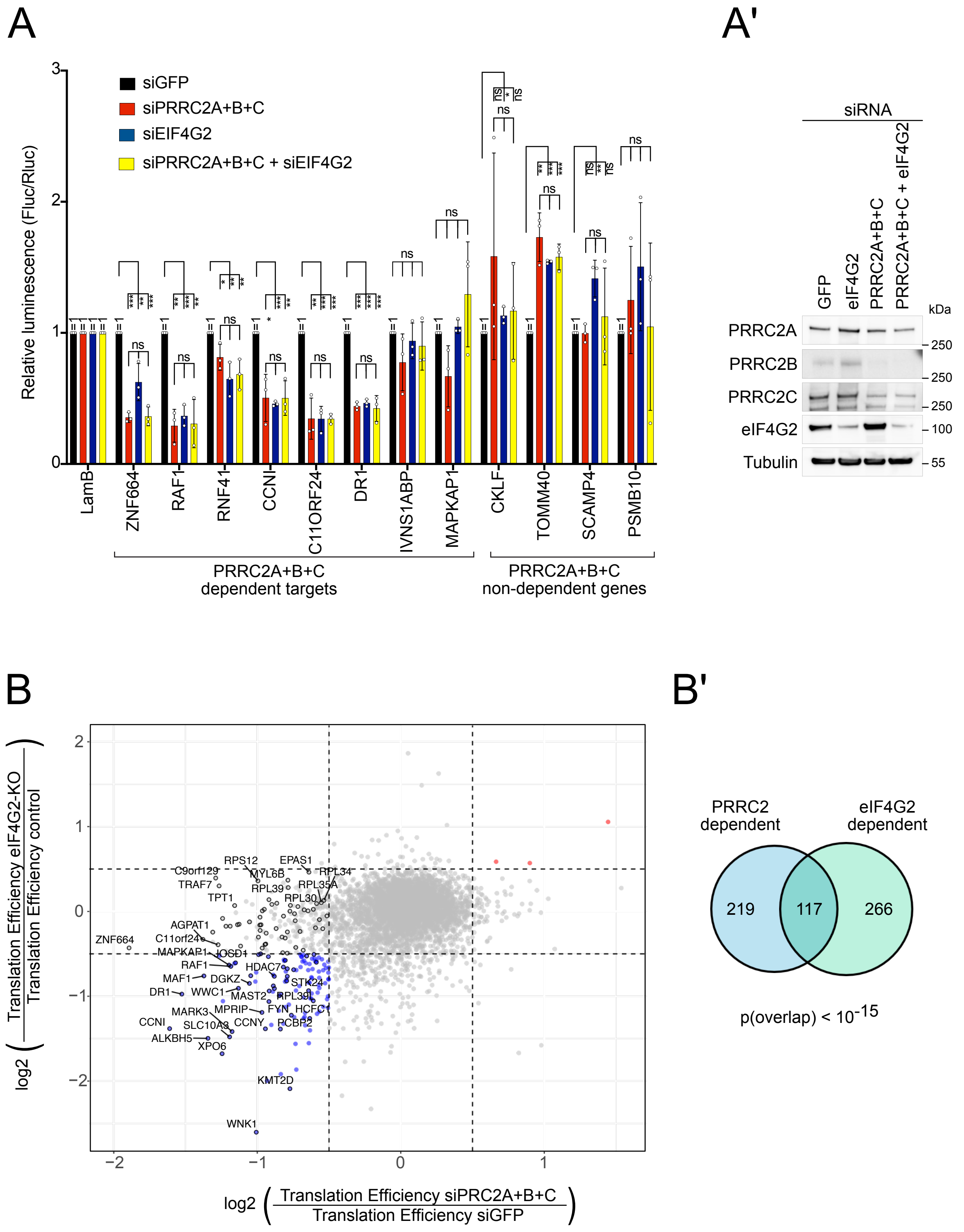

Suppl. Figure 9

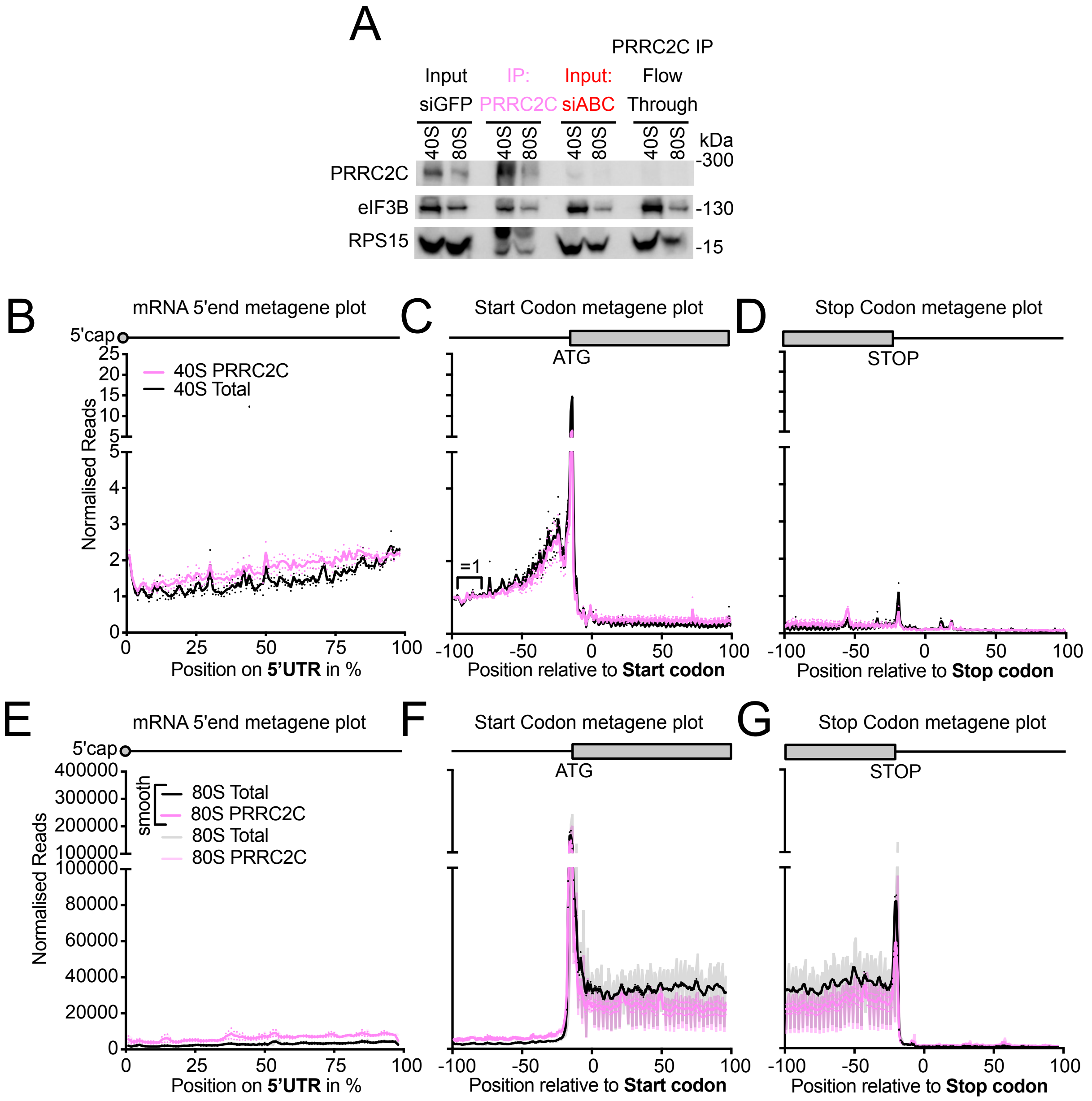

Suppl. Figure 10

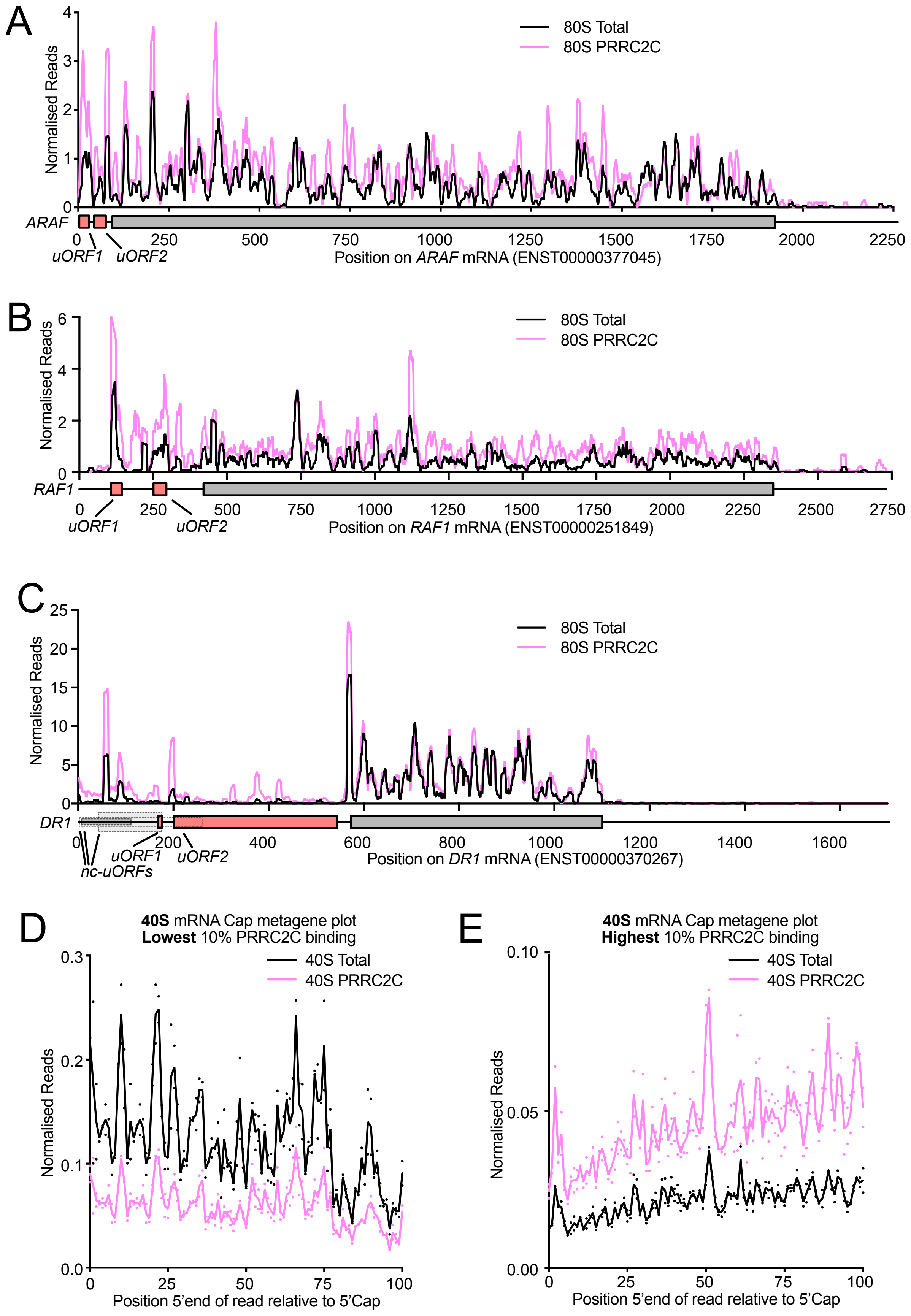

Supplement: gkad135_Supplemental_Files [file gkad135_supplemental_files.zip › Supplementary material v39+figs.pdf]
